# Supplementary material for: Tolerance Induction in Cow’s Milk Allergic Children by Heated Cow’s Milk Protein: The iAGE Follow-Up Study
Source: Nutrients. 2023 Feb 27;15(5):1181. doi: 10.3390/nu15051181 (PMC10005260; doi:10.3390/nu15051181)
Supplement: Supplementary file 1 [file nutrients-15-01181-s001.zip › Suppl S2A. Introductie koemelk flesvoeding na negatieve provocatie iAGE versie 1 29-12-2016.pdf]

Algemene Introductieregels?

- Introduceer de eerste hoeveelheid van het nieuwe voedingsmiddel vóór 12:00 uur 's morgens, zodat eventuele klachten overdag opgemerkt kunnen worden.
- Introduceer niet in situaties waarin de effecten onduidelijk kunnen zijn, zoals bij infecties, koorts, huiduitslag, opvlammend eczeem of doorkomende tanden / kiezen.
- Introduceer geen andere nieuwe voedingsmiddelen naast dit introductieschema!
- U mag de stappen eventueel iets kleiner maken en meer dagen introduceren dan aangegeven. Zorg wel dat u altijd uitkomt bij de laatste stap.
- Bij klachten zoals buikpijn en eczeem hoeft u het gebruik van het voedingsmiddel niet te stoppen. Ga terug naar de stap eerder op het schema waarbij het wel goed ging, ga hier even mee door en wacht met ophogen tot de klachten verdwenen zijn.
- Noteer bij twijfel wát u kind heeft gegeten, hoeveelheid, soort, ernst & duur van de klacht, medicatie gegeven ja/nee? Neem contact op met Kinderhaven.
- Als het geteste voedingsmiddel geen klachten heeft gegeven **moet** u het blijven gebruiken. Het is dan ook niet te verwachten dat hetzelfde voedingsmiddel later toch nog klachten gaat geven.

SUCCES!!!

Introductie Zuigelingenvoeding

Als er geen verdenking koemelkallergie is, raden we aan om gewone zuigelingenvoeding in stapjes te introduceren. Niet omdat u toch nog een reactie kan verwachten, maar meer om uw kindje langzaam te laten wennen aan een nieuwe voeding. De smaak van de voeding is namelijk ook anders, het ene kind accepteert dit sneller dan de ander.

VOORBEELD :

Dag 1
In het eerste flesje van de dag : 1 schepje hypoallergene zuigelingenvoeding vervangen door gewone zuigelingenvoeding.

Dag 2
In het eerste en tweede flesje : 1 schepje hypoallergene zuigelingenvoeding vervangen door gewone zuigelingenvoeding.

Dag 3
Al u meer flesjes per dag gebruikt : in alle flesjes 1 schepje hypoallergene zuigelingenvoeding vervangen door gewone zuigelingenvoeding.

Dag 4
In alle flesjes 2 schepjes hypoallergene zuigelingenvoeding vervangen door gewone zuigelingenvoeding.

Dag 5
In alle flesjes de ene helft hypoallergene zuigelingenvoeding en in de andere helft gewone zuigelingenvoeding.

Dag 6
Het eerste flesje van de dag geheel vervangen door gewone zuigelingenvoeding. De overige flesjes zoals stap 5; de ene helft hypoallergene zuigelingenvoeding en de andere helft gewone zuigelingenvoeding.

Na dag 6 vervangt u elke dag een flesje meer, voor een heel flesje gewone zuigelingenvoeding.

Als de gewone zuigelingenvoeding goed gaat kunt u daarna, in overleg én afhankelijk van de leeftijd van uw kindje, ook andere melkproducten gaan gebruiken zoals yoghurt en (smeer)kaas.

Na de introductie is het van belang dat uw kind blijvend én met enige regelmaat het voedingsmiddel blijft gebruiken!! Er zijn aanwijzingen dat het langdurig uitstellen van de introductie of het, na de introductie niet meer gebruiken van het voedingsmiddel, later alsnog tot een voedselallergie kan leiden.

Erasmus MC
University Medical Center Rotterdam
KinderHaven
