# Supplementary material for: Tolerance Induction in Cow’s Milk Allergic Children by Heated Cow’s Milk Protein: The iAGE Follow-Up Study
Source: Nutrients. 2023 Feb 27;15(5):1181. doi: 10.3390/nu15051181 (PMC10005260; doi:10.3390/nu15051181)
Supplement: Supplementary file 1 [file nutrients-15-01181-s001.zip › Suppl S2B. Introductie koemelk producten na een negatieve provocatie versie 1 29-12-2016.pdf]

Algemene Introductieregels

- Introduceer de eerste hoeveelheid van het nieuwe voedingsmiddel vóór 12:00 uur 's morgens, zodat eventuele klachten overdag opgemerkt kunnen worden.
- Introduceer niet in situaties waarin de effecten onduidelijk kunnen zijn, zoals bij infecties, koorts, huiduitslag, opvlammend eczeem of doorkomende tanden / kiezen.
- Introduceer geen andere nieuwe voedingsmiddelen naast dit introductieschema!
- U mag de stappen eventueel iets kleiner maken en meer dagen introduceren dan aangegeven. Zorg wel dat u altijd uitkomt op een normale consumptie; een glas melk, snee brood met (smeer) kaas of een heel bakje vla.
- Bij klachten zoals buikpijn en eczeem hoeft u het gebruik van het voedingsmiddel niet te stoppen. Ga terug naar de stap eerder op het schema waarbij het wel goed ging, ga hier even mee door en wacht met ophogen tot de klachten verdwenen zijn.
- Noteer bij twijfel wát u kind heeft gegeten, hoeveelheid, soort, ernst & duur van de klacht, medicatie gegeven ja/nee? Neem contact op met Kinderhaven.
- Als het geteste voedingsmiddel geen klachten heeft gegeven **moet** u het blijven gebruiken. Het is dan ook niet te verwachten dat hetzelfde voedingsmiddel later toch nog klachten gaat geven.

SUCCES!!!

Introductie Melk

WEEK 1

Begin met gebruik van melk(poeder) in gebakken producten zoals koekjes, brood & crackers. Bijv. melkbestanddelen in een biscuitje, kinderkoekje, krentenbol, melkbroodje, roomboter croissantje of appeltaartje. Liever hartig? Een chipje zoals Nibitt of toastje mag ook. De melk(poeder) in gebakken producten uit de oven (boven de 180 graden) wordt altijd goed verdragen. Nb: een chocolade- of crème laagje is niet meegebakken in de oven! Dit mag u pas later introduceren. Begin met een koekje en hoog de hoeveelheid op naar brood. Als dit lukt kunt u ook meerdere producten op één dag combineren.

WEEK 2

U kunt verse melkproducten gaan gebruiken. Maak pannenkoeken met melk of verwerk melk door de aardappelpuree. U kunt nu ook producten geven die niet hoog verhit zijn zoals vleeswaren met melkbestanddelen, roomboter op brood, een chocolade– of crème laagje bij biscuit. Start met het loslaten van etiketten zoals bij melkbestanddelen in sauzen, soepen e.d. Neem daarna steeds oplopende hoeveelheden, tot één melkproduct per dag.

Voorbeeld a :
Dag 1 - ¼ snee brood met (smeer)kaas.
Dag 2 - ½ snee brood met (smeer)kaas.
Dag 3 - hele snee brood met (smeer)kaas.
Voorbeeld b :
Dag 1 - paar slokjes melk of een paar hapjes danoontje / yoghurt.
Dag 2 - ½ beker melk of danoontje / yoghurt 50ml.
Dag 3 - hele beker melk, 1 groot danoontje óf een schaaltje yoghurt / vla.

U mag van de melk ook bijvoorbeeld rijstepap maken of melk toevoegen aan de flesvoeding. Voor het testen van de melk word het liefst een zo naturel mogelijk product gekozen. Zonder andere allergenen en hulpstoffen. Probeer deze week verschillende melkproducten. Als u kaas heeft geprobeerd kunt u i.p.v. kaas een keer een bakje vla geven.

WEEK 3

Hoog de hoeveelheid op tot de aanbevolen hoeveelheid per dag. Dit zijn, afhankelijk van de leeftijd, ongeveer 3 consumpties (zie tabel).

1 consumptie = 1 plak kaas van 20 gram of smeerkaas, 1 glas melk van 150 ml (bij voorkeur halfvolle melk of karnemelk en af en toe yogi of chocolademelk), 1 toetje bijvoorbeeld yoghurt, kwark of vla of af en toe andere melktoetjes zoals ijs of pudding.

Aanbeveling per leeftijd	1 - 3 jr.	4 - 8 jr.	9 - 18 jr.
Melk, melkproduct zoals yoghurt	300ml	400ml	600ml
1 plak kaas is 20 gram.	½	½	1

Let op :

Het kan zijn dat uw kind de tijd nodig heeft om aan de nieuwe smaak van melk te wennen. Na een provocatie op Kinderhaven verwachten we eigenlijk geen heftige reacties meer van melk, maar soms lukt het introduceren niet omdat de smaak van melk anders is dan de hypoallergene melk of de sojamelk. Het is voor uw kind een nieuwe smaak, daarom kan de introductie soms wat langzamer gaan dan verwacht. Als uw kindje ouder dan 1 jaar is, probeer dan de melk te introduren in de vorm van gewone melkproducten en de fles afbouwen.

Na de introductie is het van belang dat uw kind blijvend én met enige regelmaat het voedingsmiddel blijft gebruiken!! Er zijn aanwijzingen dat het langdurig uitstellen van de introductie of het, na de introductie niet meer gebruiken van het voedingsmiddel, later alsnog tot een voedselallergie kan leiden.

Erasmus MC
University Medical Center Rotterdam
KinderHaven
