# Supplementary material for: Tolerance Induction in Cow’s Milk Allergic Children by Heated Cow’s Milk Protein: The iAGE Follow-Up Study
Source: Nutrients. 2023 Feb 27;15(5):1181. doi: 10.3390/nu15051181 (PMC10005260; doi:10.3390/nu15051181)
Supplement: Supplementary file 1 [file nutrients-15-01181-s001.zip › Supplement S1 Protocol iAGE study version 3.0 dd 23072018.pdf]

Clinical Trial Protocol

Immunogenic Properties of Heated and Glycated Cow's Milk Protein – Effect on Resolution of Cow's Milk Allergy (iAGE study)

Protocol ID: iAGE (NL61774.078.17)

Short title: Immunogenicity of heated and Advanced Glycated cow's milk End-products (iAGE study)

Version: 3.0

Date: 23-July-2018

Coordinating investigator: N.W. de Jong PhD, Department of Internal Medicine, Section Allergology gK 323, Erasmus MC, PO Box 2040, 3000 CA Rotterdam, the Netherlands, +31 6 2169 7954, n.w.dejong@erasmusmc.nl

Participating sites:

Canisius-Wilhelmina Ziekenhuis, Nijmegen

Catharina Ziekenhuis, Eindhoven

Elkerliek Ziekenhuis, Helmond

Erasmus MC, Rotterdam

Martini Ziekenhuis, Groningen

Noordwest Ziekenhuisgroep, Alkmaar

Onze Lieve Vrouwe Gasthuis, Amsterdam

UMC Groningen

Zuyderland MC, Heerlen

Co-investigators:

K.A. Hettinga PhD, Wageningen University, the Netherlands

R. Gerth van Wijk, MD PhD, Erasmus MC Rotterdam, the Netherlands
H.J. Wichers PhD, Wageningen University, the Netherlands

H.F.J. Savelkoul PhD, Wageningen University, the Netherlands

A.B. Sprikkelman, MD PhD, UMC Groningen, the Netherlands

Sponsor: Erasmus MC, Rotterdam

Subsidising Party: NWO-TTW

Independent Physician: K.F.M. Joosten, MD PhD, pediatic-intensivist, Erasmus MC, Department of Pediatrics

Laboratory sites: Wageningen University and Research Centre

Pharmacy: N.A.

PROTOCOL SIGNATURE SHEET

Name	Signature	Date
Head of Department Erasmus MC:<br>Prof. dr. R. Gerth van Wijk		**23-07-2018**
Coordinating Investigator/Project leader/Principal Investigator:<br>Dr. N.W. de Jong		**23-07-2018**

Table of contents

SUMMARY 7
1 INTRODUCTION AND RATIONALE 9
1.1. Cow's milk allergy; induction and tolerance 9
1.2. Conflicting effects of processing of milk on allergenicity and/or immunogenicity 9
1.3. Objectives and approach 10
2. STUDY OBJECTIVES 11
2.1. Primary objectives 11
2.2. Secondary objective 11
2.3. Aim of the study 11
3. STUDY DESIGN 12
3.1. Study design 12
3.2. Follow-up and tolerance measurement 12
4. STUDY POPULATION 13
4.1. Population 13
4.2. Inclusion criteria 13
4.3. Exclusion criteria 13
4.4. Sample size calculation 13
5. TREATMENT OF SUBJECTS 14
5.1. Investigational treatment 14
5.1.1. Start of treatment 14
5.1.2. Added amount, dosing and feeding plan 14
5.2. Use of co-intervention 15
5.3. Escape medication 15
6. INVESTIGATIONAL PRODUCT 16
6.1. Name and description of investigational product(s) 16
6.2. Summary of findings from non-clinical studies 16
6.3. Summary of findings from clinical studies 16
6.4. Summary of known and potential risks and benefits 16
6.5. Description and justification of route of administration and dosage 16
6.6. Dosages, dosage modifications and method of administration 17
6.7. Preparation and labelling of Investigational Medicinal Product 17
6.8. Drug accountability 17
7. NON-INVESTIGATIONAL PRODUCT 18
7.1. Name and description of Non Investigational product(s) 18
7.2. Summary of findings from non-clinical studies 18
7.3. Summary of findings from clinical studies 18
7.4. Summary of known and potential risks and benefits 18
7.5. Description and justification of route of administration and dosage 18
7.6. Dosages, dosage modifications and method of administration 18
7.7. Preparation and labelling of Non Investigational Medicinal Product 18
7.8. Drug accountability 18
8. METHODS 19
8.1. Study parameter(s)/endpoint 19
8.1.1. Main study parameter/endpoint 19
8.1.2. Secondary study parameters/endpoints 19
8.1.3. Other study parameters (if applicable) 19
8.2. Randomisation, blinding and treatment allocation 19
8.3. Visit schedule and study procedures 19
8.3.1. Medical history parents and child other baseline characteristics 20
8.3.2. Subject diary. 20
8.3.3. FAQLQ Questionnaire 20
8.3.4. Skin prick tests 21
8.3.5. Blood samples 21

8.3.6. Double blind placebo controlled food challenge 21
8.3.7. Microbiome measurements 24
8.3.8. Regular telephone calls throughout the study 24
8.4. Withdrawal of individual subjects 24
8.5. Replacement of individual subjects after withdrawal 24
8.6. Follow-up of subjects withdrawn from treatment 25
9. SAFETY REPORTING 26
9.1. Temporary halt for reasons of subject safety 26
9.2. AEs, SAEs and SUSARs 26
9.2.1. Adverse events (AEs) 26
9.2.2. Serious adverse events (SAEs) 26
9.2.3. Suspected unexpected serious adverse reactions (SUSARs) 26
9.3. Annual safety report 26
9.4. Follow-up of adverse events 26
9.5. Data Safety Monitoring Board (DSMB) 27
10. DATA COLLECTION AND STATISTICAL ANALYSIS 28
10.1. Primary objective 28
10.2. Secondary objectives 28
10.3 Other study parameters 28
10.4 Interim analysis 28
11. ETHICAL CONSIDERATIONS 29
11.1. Regulation statement 29
11.2. Recruitment and consent 29
11.3. Minors 29
11.4. Benefits and risks assessment 29
11.4.1. Burden/risks: 29
11.4.2. Adverse Events 30
11.4.3. Additional Safety Monitoring and Risk Mitigation Details 30
11.4.4. Data and Safety Monitoring Plan 31
11.4.5. Potential Benefits 31
11.4.6. Importance of the Knowledge to be gained. 31
11.4.7. Risk assessment 31
11.4.8. Overall risk-benefit statement 32
11.5. Compensation 32
11.6. Incentives 32
11.7. Storage of and access to study materials 32
12. ADMINISTRATIVE ASPECTS AND PUBLICATION 34
12.1. Handling and storage of data and documents 34
12.2. Monitoring and Quality Assurance 34
12.3. Amendments 34
12.4. Annual progress report 34
12.5. Temporary halt and (prematurely) end of study report 34
12.6. Public disclosure and publication policy 35
12.7. Existing infrastructure 35
13. STRUCTURED RISK ANALYSIS 36
13.1. Potential issues of concern 36
13.1.1. Level of knowledge about mechanism of action 36
13.1.2. Previous exposure of human beings with the test product and/or products with a similar biological mechanism 36
13.1.3. Can the primary or secondary mechanism be induced in animals and/or in ex-vivo human cell material 36
13.1.4. Selectivity of the mechanism to target tissue in animals and/or human beings 36
13.1.5. Analysis of potential effect 36
13.1.6. Pharmacokinetic considerations 36
13.1.7. Study population 36

13.1.8. Interaction with other products 36
13.1.9. Predictability of effect 36
13.2. Synthesis 36
14. REFERENCES 38
Appendix 01 – Flow chart 40
Appendix 02 – Study visits and assessments 41
Appendix 03 – Questionnaire FAQLQ 42
Appendix 04 – Recipe challenges CM various kits 48
Appendix 05 – Dosing of product 51
Appendix 06 – Questions bi-monthly telephone call 52
Appendix 07 – Diary for parents 54
Appendix 08 – Skin prick test form 56
Appendix 09 – Types of formulas in NL 57
Protocol amendment 01 – side study goats milk sensitization in cow's milk allergic infants..58
Protocol amendment 02 – side study microbiome in cow's milk allergic infants 60

LIST OF ABBREVIATIONS AND RELEVANT DEFINITIONS

AGE	Advanced Glycated end Products
BAT	Basophil Activation Test
BP	Birch Pollen
BMT	Baked Milk Tolerant
BMR	Baked Milk Reactive
CCMO	Centrale Commissie Mensgebonden Onderzoek (Dutch competent authority)
CH	Case History
CM	Cow's Milk
CMA	Cow's Milk Allergy
CRD	Component Resolved Diagnosis
DBPCFC	Double Blind Placebo Controlled Food Challenge
DSMB	Data Safety Monitoring Board
EAACI	European Academy of Allergy and Clinical Immunology
eHF	Extra Hydrolysed formula
ELISA	Enzyme Linked Immuno solvent Assay
ESFA	European Food Safety Authority
FA	Food Allergy
FAQLQ-PF	Food Allergy Quality of Life- Questionnaire- Parent Form
GP	Glycated Cow's milk Protein
GP	General Practitioner
GrP	Grass Pollen
FcεR	Fcε-receptor
HEP	Histamine Equivalent Prick
HDM	House Dust Mite
IFT	Infant and Toddlers
IgE	Immuno globuline E
LOAEL	Lowest Observed Allergic Event Level
Ml	Millilitre
MRA	Mediator Release Assays
MRP	Maillard reaction Products
NOAEL	No Observed Allergic Event Level.
NVK	Nederlandse Vereniging voor Kindergeneeskunde
OFC	Oral Food Challenge
QA/QC	Quality assurance/ Quality control
SPT	Skin Prick Test
TIS	Three Item Severity Score
UP	Unheated Protein
UMCG	University Medical Centre Groningen
WUR	Wageningen University

SUMMARY

Rationale: Cow's milk allergy (CMA) is the most common food allergy in young children, with prevalence rates estimated in the range of 2.0% of all children. The form in which milk proteins are delivered to CMA children determines the induction of allergic symptoms or tolerance ("outgrowing" the allergy), with spontaneous tolerance development in about half the CMA children. Processing of cow's milk (CM) determines the immunogenic and allergenic properties of the milk proteins and is therefore implicated in induction of allergy as well as the induction of tolerance. Literature analysis indicates that there are variable effects of processing of milk on allergenicity and/or immunogenicity. After CMA has developed, consumption of 'baked' milk products (i.e. milk-containing bakery products) can lead to accelerated resolution of CMA.

Primary objective: Develop and test a prototype product containing extensively heated glycated CM protein, to accelerate the resolution of CMA in children

Study design:
Double-blind randomised placebo-controlled therapeutic multicenter national intervention study. Non-drug study. Double-blind randomized food challenge. Duration per patient approximately 2 years.

Study population:
The aim is to recruit at least 200 participating children (age 0-2 years) suspected of CMA, confirmed with a positive double blind placebo controlled food challenge (DBPCFC) with CM. Children will be randomised either in the Glycated protein (GP) group or the Placebo group. Approx. 12 clinics, the 'IAGE study group', will include the subjects and will carry out the clinical part of the study:

Study interventions:
- Total study duration: 2 years (or until development of tolerance);
- 7 visits (months 0, 4, 8, 12, 16, 20, 24);
- 6 phone calls to parents (between 24 and 48h after each challenge);
- 4 physical examinations (months 0, 8, 16, 24);
- 1 buccal swab (for genetic testing) from mother and child;
- 3-4 double blind placebo controlled food challenges (DBPCFC), each DBPCFC consists of 2 challenges (1 placebo, 1 verum, 1 week apart, randomized): during screening (in most clinics this challenge is part of standard care, in line with current guideline) and months 8, 16, 24 (in some clinics regular follow-up challenges are part of standard care as well, however for this study these 3 DBPCFC are considered study interventions);
- 4 skin prick tests (SPTs) (months 0, 8, 16, 24). Each SPT will consist of 5 pricks (CM, GP, 2 positive and 1 negative control);
- 4 blood samples (months 0, 8, 16 and 24 (or when tolerance has developed), blood volume: <6 months of age 1 finger prick per occasion, ≥6 months of age 4 ml;
- 4 stool samples (months 0, 8, 16, 24), microbiome testing.
- completion of questionnaires (months 0, 8, 16 and (FAQLQ only) 24), medical history child, parents, quality of life (FAQLQ);
- interventional product: GP or placebo to be added to the regular daily nutrition of the subject starting at the first study day.

Nature and extent of the burden and risks associated with participation, benefit and group relatedness:

- See study interventions above.
- The children will receive the normal treatment, drink the formula that is prescribed by the first or second line medical care. The standard care will not be changed in both groups.
- The burden and risks will be limited due to extensive experience with DBPCFCs. However, the outcome of the DBPCFC remains unpredictable and reactions may occur. Therefore, the DBPCFC will take place at the children's department, at the medium care unit, equipped with all necessary technical and medical emergency facilities.
- The study can only be performed in this group of children because high numbers of CMA are found in very young children.
- The DBPCFC is the only available diagnostic tool.
- Furthermore, this group is prone to develop tolerance and the window of tolerance induction is present in the first 2 years of the child.
- The above-described method is the golden standard for the diagnosis of CMA in children, according to the Dutch guidelines.(18) The DBPCFC is performed for an additional 3 times in order to measure any tolerance to CM during the intervention period.

Potential benefits:

Potential benefits are:

1. Subjects may be desensitized to CM and have a reduced risk of anaphylaxis from an accidental ingestion of CM proteins.
2. Subjects may become tolerant to CM and do not have to avoid it.

1 INTRODUCTION AND RATIONALE

1.1. Cow's milk allergy; induction and tolerance

Cow's milk allergy (CMA) is the most common food allergy in young children, with prevalence rates estimated at 2.0% (range 0.1-4.2%) in children 0-1 years old. (1) For this research project CMA is defined as double-blind, placebo-controlled, challenge-proven allergy to cow's milk (CM), independent of IgE-status. In approximately 50% of the children, CMA spontaneously resolved over 66 months of follow-up. Baseline milk-specific IgE level, SPT wheal size, and eczema severity are important predictors for the likelihood of resolution.(2) Nevertheless, in a substantial part of the children the allergy is not IgE –mediated. The form in which oral allergens are delivered to CMA children determines either the induction of milk-specific allergic symptoms or the induction of tolerance ("outgrowing" the allergy). Processing methods of CM determine the immunogenic and allergenic properties of the milk proteins and are therefore implicated in the distinctive consequences of milk exposure: induction of allergy as well as the induction of tolerance. Modification of potentially allergenic epitopes, alterations of the food matrix, induction of Maillard reaction products, hydrolysis and other forms of food processing all modulate the allergenic and immunogenic properties of allergenic proteins present in milk.

1.2. Conflicting effects of processing of milk on allergenicity and/or immunogenicity

Heating-induced alterations in CM proteins may promote early development of allergic inflammation and/or sensitisation stage of CMA pathology. (3) For instance, cross-linking of ß-lacto globulin (BLG), as a consequence of pasteurisation, led to intestinal uptake via Peyers patches rather than via intestinal uptake, and in vitro exposure of dendritic cells (DC) to cross-linked BLG initiated a Th2-skewing response of these DCs.(4, 5) Earlier, it has been recognised that exposure of DCs to allergens predisposes these DCs to initiate a Th2-like response. Apparently, the type of heating that is applied during industrial processing of milk (i.e. time and temperature of treatment, e.g. pasteurization/sterilization, evaporation, drying) and the presence of reducing sugars are important for its eventual immunogenicity/allergenicity. This is especially relevant because milk contains high lactose levels.

In contrast, consumption of 'baked' milk products (i.e. bakery products containing milk, like muffins) can lead to accelerated resolution of CMA. Previous research showed the accelerated resolution of CMA in a majority (ca. 75%) of CMA children, when they are exposed to such products containing 'baked' milk, in comparison to untreated controls. Apparently, upon 'baking' the milk, compounds are formed that aid in resolving established, clinical CMA.

No immunological mechanisms underlying these observations have been reported so far. Products used for these studies have never been well characterized, neither from physical-chemical nor from in-vitro immunological perspective. Furthermore, there appears to be heterogeneity in the CMA population with respect to type of allergy (transient vs. persistent CMA) as well as sensitivity to altered milk proteins leading to different clinical symptoms of allergy. This has been related to consumption of heated milk protein-containing products and termed; baked milk tolerant vs. resistant (BMT & BMR), with BMT but not BMR being associated with transient CMA.(6)

Exposure to CM is mandatory for the development of CMA. Across the EU, there is considerable variation in the extent of thermal processing of CM for human consumption, dependent on target groups, national regulations, etc. Generally, CM for infant formula is strongly thermally processed to obtain a microbiologically safe product. Side-effects of this strong thermal processing are denaturation, aggregation, and gyration, (i.e. undergo Maillard reaction) of proteins, the latter potentially resulting in Advanced Glycation End-products (AGE). Either of these structural changes may lead to activation of the innate arm of intestinal immunity through particular receptors (RAGE, CD36, SR-AI/II, Mannose receptor, and lectins like Dectin-1 and Galectin-3 (7, 8). Apparently, consumption of differently thermally processed milk

can have differential effects on the initiation and/or development of CMA, and the resolution of established CMA.

1.3. Objectives and approach

In this proposal the tolerance inducing properties of GP products in CMA children will be investigated.

The following hypothesis will be subject in the study:

Maillard reaction products (MRPs) (e.g. Advanced Glycation End- Products [AGE] products) formed upon heat processing of milk can, in children with pre-existing CMA, induce immune tolerance

Primary objective: Design, study, and test a prototype product based on extensively heated glycated CM protein, to accelerate the resolution of CMA. To corroborate the predicted clinical tolerance induction, usability, safety and efficacy will be measured.

It has been described in literature that severely heated milk proteins in a bakery-like matrix ('baked') are causally involved in the accelerated resolution of established CMA. We propose an integrated multidisciplinary research approach. To reach the project goals, a combination of research focusing on a mechanistic understanding of the effects of heating on milk proteins and subsequent testing of the tolerance inducing capacities of the product in CMA patients is needed. As heating has differential effects, (in presence of sugar: glycation), we will study this protein type in this research: glycated protein (GP) versus placebo.

First, a characterization of this proteins type is needed to characterize the chemical compounds formed during the applied processes.

Second, the immunological response to the milk protein needs to be established.

Finally, the physical-chemically and immunologically characterized milk protein will be tested, focusing specifically on tolerance induction.

Societal objectives, relevance & impact: The overall beneficial effects of treating and/or preventing CMA on quality of life and health economics will be:

- Reducing the number of children that develops CMA
- Developing diagnostic markers and methods to estimate efficacy of treatment of CMA
- Lowering the medical costs in children caused by their CMA e.g. doctors' visits, hydrolysed formula diet.
- Preventing the child form entering the "allergic march", reducing healthcare costs and improving quality of life on the short term as well as on the long term.

2. STUDY OBJECTIVES

2.1. Primary objectives

Develop and test a prototype product containing extensively heated -glycated CM protein, to accelerate the resolution of CMA in children.

2.2. Secondary objective

1. Processing guidelines for preparation of less immunogenic/allergenic yet microbiologically safe milk products (target group: infants),
2. Understanding the mechanism of how the immunomodulatory active processed milk products/ingredients (GP-containing products):
   a. Exert endogenous tolerogenic activity, usable for reduction of incidence of CMA,
   b. Induce tolerogenic activity, hypothetically as MRPs, useful to accelerate resolution of full-blown clinical allergy,
3. Protocols, GP-products, for desensitization treatment,
4. Genetic markers associated with CMA will be identified,
5. Measure effects on the microbiome of the child before and after the intervention.

2.3. Aim of the study

The aim of this study is to measure the tolerance inducing properties of GP products in CMA children, which should accelerate tolerance induction to normally processed milk. In earlier studies CMA children were subdivided into a large (~75%) group of BMT and a smaller (~25%) group of BMR patients. (9) Since no universal agreement exists regarding the optimal recipe to use for heated milk challenges, till now baked muffins or waffles or pizzas were used to divide the children in these two groups, which is rather inappropriate. (10) In contrast, using products with exact known heated glycated protein concentrations, allows us to discriminate children that are tolerant or reactive, to glycated proteins (GP) in a more standardised way, which may even give complete different BMT/ BMR ratio.

As the majority of children outgrow milk allergy at a very young age, the study will focus on CMA-children from the day they are diagnosed with CMA. (11, 12) In earlier studies, Kim et al. (8) found that the window of tolerance induction and/ or outgrowing CMA in BMT children is between the age of 12 and 24-36 months (30%-48% resp.). Using this window, the children will have increased benefit from early intervention via immune modulatory strategies. (10) To distinguish GP-tolerant and -reactive children, a broad scale of tools can be used. (13, 14) Until now, no thresholds are suggested to discriminate transient or persistent CMA children. Therefore, the use of a double-blind, placebo-controlled food challenge (DBPCFC) is still necessary. (15) These challenge data will be linked to clinical chemical data, to develop diagnostic and prognostic tools to classify transient or persistent CMA children. Furthermore, genetic polymorphisms, which show association with clinical reactivity to cow's milk, will be identified (16). They can be of significant value to predict the risk of CMA and give insight into the mechanisms leading to clinical reactivity to cow's milk. In recent findings food allergy appear to be associated with alterations in the gut microbiota or dysbiosis early in life, that may be predictive of disease persistence versus tolerance acquisition (17). We therefore aim to measure changes in the microbiome of the patient during this intervention study.

3. STUDY DESIGN

3.1. Study design

Double-blind randomised placebo-controlled therapeutic multicenter national intervention study. Non-drug study.

The aim is to recruit at least 200 (100 per study arm) participating children (age 3 months – 2 years.) with a proven CMA, e.g. positive DBPCFC to CM (22). In this study this number should be sufficient to measure tolerance in 8, 16 or 24 months (see paragraph 5.3 for the sample size calculation). We hypothesize that 1.500 subjects with symptoms possibly related to CMA must be screened in order to randomize 200 subjects with a DBPCFC positive for CM. This is based on earlier prevalence studies: ±15-20% positive in challenges e.g. the Europrevall studies **(**perceived CMA in a European population 18-20%, DBPCFC proven only 1,0- 2,2 – 2,8%) (23-25).

It should be feasible to recruit 1.500 subjects in 18 months in 10-12 hospitals. This equals approx. 150 DBPCFC in 18 months (8.3 per month). This should result in approx. 22 CM positive DBPCFC per clinic in 18 months (220 in total). Taking into account a drop-out rate of 10%, results in a total of 200 completed cases based on a recruitment period of 18 months.

According to the standard care, a child suspected of having CMA will be referred for a DBPCFC with CM in the formula the child daily drinks (initial DBPCFC). In case of a positive DBPCFC for CM, the parents will be asked for participation for the child in the study. The child enters the study at visit 1; T=1 and after informed consent, several questionnaires, physical examination, SPT, and blood sample will be performed.

In total the subjects will visit the hospital 7 times. During the first visit both consenting parents (or legal guardian) will sign the informed consent first. Thereafter study procedures may be carried out.

3.2. Follow-up and tolerance measurement

The follow-up comprises visits at 8 months, 16 months and 24 months in which DBPCFC's with cow's milk will be performed to measure tolerance. Questionnaires, SPTs and blood samples will be a part of the follow-up visits. In case the child reacts positive in the challenge, they remain in the study and continue using the same product in the same study arm. Depending on the weight of the child, the product will be up dosed. In case the child is tolerant to normal CM, the child will stop the intervention. They will be advised to introduce CM into the diet and will receive an introduction schedule to guide this. After introduction they will be advised to use CM at home regularly. The CM tolerant children will enter a follow-up program consisting of regular telephone consults, using specific questionnaires on diet and allergic status. For the children that react positive in the 8,16 and 24 months challenges, intervention stops at 24 months.

4. STUDY POPULATION

4.1. Population

Children with CMA will be included according to the latest developed Dutch National Guideline (18).This guideline recommends aDBPCFC, in case a CMA is suspected. The children with a positive challenge will be asked to participate in the study. Inclusion comprehends: questionnaires on history, SPT's, DBPCFC's,blood sample and stool analysis. In the first phase of the study, the child will be treated as according to the national guideline (CM-free diet and administration of the extra hydrolysed formula (eHF) in case of failure of breastfeeding). Extensive dietary advice on the cow's milk free diet will be given to the parents and lists with products containing CM proteins will be distributed. Parents will have regular telephone consultations (every 2 months) on adherence to the product and the CM free diet.

4.2. Inclusion criteria

All children (3 months up to and including 2 years) referred to the hospital with a suspected CMA and a positive DBPCFC for CM, in line with the Dutch CM guideline. (18)

- Age: 3 months up to 2 years.
- Children with a positive DBPCFC with CM. This is defined as a well-documented DBPCFC with objective symptoms <2 hours that was performed ≤4 weeks prior to inclusion in the study.
- Written informed consent by parents or legal guardian.

4.3. Exclusion criteria

- Current participation or participation in the last 12 months in a medical scientific interventional study for food allergy and/or with an investigational new drug.
- Clinically significant immunological, cardiovascular or malignant disease.
- Inability to discontinue antihistamines for skin testing and oral food challenges.
- Caregiver not able to read and understand the Dutch language.
- Caregiver not able or willing to comply with the study procedures.

4.4. Sample size calculation

The primary objective focuses at the effect of the desensitization treatment on CM tolerance at 24 months. Based on a previous study, it is expected that 33% of children in the control group will be CM tolerant after 24 months and in the intervention group 56%. (6) Using an alpha of 0.05 (2-sided) and 90% power, 93 children per group will be required.
It is expected that for the recruitment of 186 children with a positive DBPCFC forCM approx. 1500 children must be screened, see paragraph 4.1 for the feasibility analysis of recruitment.

5. TREATMENT OF SUBJECTS

5.1. Investigational treatment

5.1.1. Start of treatment

The test product (GP or placebo) will be delivered to the clinic within one week after randomisation. The placebo consists of glucose. The volume and doses are calculated and based on the maximum daily intake of the GP product, depending on the weight of the subject (0.8 to 1.4 grams of GP product). For placebo the same amount will be added.

At Visit 1 the first dose of the GP or placebo product will be added to the subject's own regular eHF formula, in presence of the parents, also for learning purposes.

First intake of the product will take place in the hospital in incremental doses. Between doses the child will be observed for possible symptoms for 30 minutes. Introduction will take place using an introduction schedule with incremental doses, adjusted according to EAACI Guidelines for DBPCFC (31):

	Doses mg							Cum
GP product (mg)	3	10	30	100	300	1000	2000	3443
ml eHF	0,3	0,6	1,7	5,8	17,4	58,1	116,3	200

The parents receive enough product for 4 months, together with instructions how to add the weight dependent dose of the product to the daily diet of the subject. The parents will receive a container with a small spoon with the exact size to fit the correct dose of the product. The product can be added to a bottle with milk formula or another product like for example porridge or yoghurt.

Parents will bring the container back to the hospital at the next visit. The container will be weighed before and after use to check for compliance.

5.1.2. Added amount, dosing and feeding plan

The product is added with a small spoon to the child's standard formula. A weight dependent schedule will be distributed to the parents with the volume that should be added to one portion of the daily used eHF of the child. An instruction form is added to the product. The first intake of the product takes place at V=1 at the hospital, in up dosing amounts, see Appendix 2 (Visit overview).

The added amount of product has been calculated based on the table below.

The placebo will consist of glucose in powder form.

The test product will be supplied in bulk containers with a scoop of the proper size. The right amount of test product needs to be added to the first bottle or another soluble food, e.g. soy-yoghurt or porridge. Parents will be supplied with sufficient test product for 4 months. Parents will receive new test product 3 times in between study visits. The containers can be stored at room temperature, but must be stored at 4° C when opened.

Child		Total protein intake (basis: 2 gr/kg)	Protein via study material (calculated)	Protein via study material (actual)
Age (months)	Weight (kg)	gr/day	Grams/day	Amount (g)
3	6	12	0.6	0.8
6	7	14	0.7	0.8
9	8	16	0.8	0.8
12	9	18	0.9	1.1
15	10	20	1	1.1
18	11	22	1.1	1.1
21	12	24	1.2	1.4
24	13	26	1.3	1.4
30	14	28	1.4	1.4
36	15	30	1.5	1,7
48	17	34	1,7	1,7

5.2. Use of co-intervention

The subject remains on a CM-free diet throughout the study. There are no other diet restrictions. At Visit 1 advice on the CM-free diet will be given to the parents. They will be given a list of products containing CM proteins. A specialized allergy nurse will call the parents on a two monthly basis for dietary consultation, see Appendix 6 (Bimonthly telephone questions). Questions on diet, accidental CM intake and symptoms will be asked and a short repost will be added to the subject CRF.

5.3. Escape medication

Parents may receive an adrenaline auto-injector upon investigator’s decision, based on the severity of the reaction(s).

6. INVESTIGATIONAL PRODUCT

6.1. Name and description of investigational product(s)

Heated glycated cow's milk protein (GP product).

6.2. Summary of findings from non-clinical studies

N.A.

6.3. Summary of findings from clinical studies

Findings from clinical studies are reviewed by Leonard et al. (33).

Summary: Extensively heated (baked) milk and egg are tolerated by most of the children with IgE-mediated allergy to unheated milk and egg. Tolerance of baked milk and egg precedes tolerance of the unheated. milk and egg and is a marker of a less severe and less persistent allergy, especially regarding CM allergy. Current evidence favors incorporating baked milk and egg into the diet of children who can tolerate them because it appears to be safe, to be well tolerated, and to accelerate development of tolerance to unheated milk and egg. If parents cannot reliably report a history of regular tolerance of baked milk or egg at home, physician-supervised OFCs are advised for an initial introduction of baked milk and egg.

Furthermore S.Cherkaoui et al. (34) has performed a study with baked milk powder and conluded that: "Challenge with instant skim milk powder could be a safe, convenient and easily standardisable alternative to home baked food for heated milk challenge. Further controlled studies are needed before this can be implemented to practice.

6.4. Summary of known and potential risks and benefits

From the same review article from Leonard et al. we quote the following:

Regular ingestion of baked milk and egg was found to be well tolerated in children without changes in underlying allergic diseases, growth, or intestinal permeability. No serious reactions were reported with regular ingestion of baked milk or egg at home in the prospective clinical trials. After a successful baked milk or egg challenge, detailed instructions on how to add baked milk or egg at home are vital.
Because there are no biomarkers for tolerance of less extensively heated proteins at this time and significant reactions have occurred to these forms, there is still a need for supervised OFCs to assess the tolerance to these foods."
Another study from S.Cherkaoui et al. Allergy Asthma Clin Immunol (2015) 11:39 reported the following sentence:
In heated milk reactive patients, challenge reactions were mostly mild, except for one child who required epinephrine administration. In patients who tolerated the heated milk challenge, there was no serious reaction upon reintroduced at home. The dose of milk protein reached with this protocol (4 g) thus appears to allow a safe reintroduction of heated milk products at home.
The dose in this study was considerably higher (4grams) than the dose in the iAGE study

6.5. Description and justification of route of administration and dosage

The test product will be supplied in bulk containers with a scoop of the proper size. The right amount of test product needs to be added to the first bottle or another soluble food, e.g. soy-yoghurt or porridge. Total amount of added protein to the daily intake will not exceed the 5 %.

6.6. Dosages, dosage modifications and method of administration

The volume and doses are calculated and based on the maximum daily intake of the GP product, depending on the weight of the subject (0.8 to 1.4 grams of GP product).

6.7. Preparation and labelling of Investigational Medicinal Product

N.A.

6.8. Drug accountability

The containers with the product will be weighed upon delivery to the parents and upon return to the study team.

The product will be transported in cooled containers.

7. NON-INVESTIGATIONAL PRODUCT

7.1. Name and description of Non Investigational product(s)

N.A.

7.2. Summary of findings from non-clinical studies

N.A.

7.3. Summary of findings from clinical studies

N.A.

7.4. Summary of known and potential risks and benefits

N.A.

7.5. Description and justification of route of administration and dosage

N.A.

7.6. Dosages, dosage modifications and method of administration

N.A.

7.7. Preparation and labelling of Non Investigational Medicinal Product

N.A.

7.8. Drug accountability

N.A.

8. METHODS

8.1. Study parameter(s)/endpoint

8.1.1. Main study parameter/endpoint

- Tolerance development.

8.1.2. Secondary study parameters/endpoints

- FAQLQ Questionnaire
- IgE.

8.1.3. Other study parameters (if applicable)

- Skin Prick Test
- Blood samples
- Microbiome measurments
- Diary
- Genetic polymorphism.

8.2. Randomisation, blinding and treatment allocation

Subjects are given a unique code number, a combination of the center number (see paragraph 4.4) and a sequential number, e.g. the first subject from center 111 will be given the code number 111001.

Randomisation, transportation and distribution of the product will be the responsibility of the Erasmus MC. Ordering of the product for new study subjects will be done as soon as possible AND at latest immediately after the 48 h telephone call of Visit 1. Randomization will take place at the same time.

8.3. Visit schedule and study procedures

The standard protocol for the confirmation or rejection of CMA is a DBPCFC. This challenge must be performed at an experienced medical center.
One of the inclusion criteria for the iAGE study is a positive DBPCFC for CM. Normally, a documented positive DBPCFC for CMA will already available from the standard medical practice. So the DBPCFC need not be repeated for study purposes. Existing data may be used, provided that it is well documented, was performed at most 4 weeks prior to inclusion in the study and gave objective symptoms within 2 hours.

In the context of standard medical practice, parents will be informed about the result of the DBPCFC of their child. In case of a positive DBPCFC they will be informed about the iAGE study during the same conversation as well. Interested parents will be given the informed consent form about the iAGE study for reading. The physician asks for permission to have the parents called by a member of the study staff 2 weeks later. During this contact the member of the study staff will ask the parents about their interest for participation of their child in the iAGE study.

Interested parents will be invited to the nearest hospital for the full informed consent procedure. Parents willing to have their child participate, will be asked to sign the informed consent form. Thereafter study procedures may be initiated. Parents will be given up to 14 days to consider their decision.

Baseline tests, e.g. medical history, physical examination, SPT, blood and stool sample, FAQLQ (quality of life questionnaire) will be performed on the first treatment day of the study (see Appendix 1).

After randomization, the first intake of the GP or placebo product will be given to the subject in the hospital. For safety reasons the GP and placebo product will be given with an introduction schedule with incremental amounts of CM protein, adjusted according to the EAACI Guidelines for DBPCFC. During Visit 1 the product will be prepared and administered by the parents, supervised by the research nurse and in presence of the physician. After a successful introduction at Visit 1, the product will be provided to the parents in a volume sufficient for 4 months.

Eight, sixteen and twenty-four months after the start of the product administration, a DBPCFC will be performed. The CM protein doses of the challenge material must be equal to the screening DBPCFC. During the study subjects may change their present formula to another eHF formula, soy milk or rice milk. Consequently, for the next DBPCFC, the formula must be adapted to the formula the child is using at that time, however the protein doses of the challenge material (CM) must be the same. Phone calls (to enquire about any delayed symptoms) will be made between 24 and 48h after each challenge. During this telephone call the parents will be informed about the result of the DBPCFC. All assessments to be performed are listed in Appendix 2.

When a new challenge remains positive, the subject remains in the study (in the same study arm). The dose of the test product will be increased according the actual weight after each visit. When an initially positive challenge becomes negative, the subject is declared tolerant to CM and will exit the study.

New weight dependent product will be given to the parents at visit 1a, 3a, 5a (after 4, 12 and 20 months

8.3.1. Medical history parents and child other baseline characteristics

A questionnaire will be used to assess the medical history of mother, father and child at Visit 1. Data collected will include date of birth, sex, race, ethnicity, height and weight, relevant medical history and current medical conditions, prior and concomitant medications, delivery method, breastfeeding, diet of the mother, smoking, vitamin intake, atopy.

8.3.2. Subject diary.

The parents of the subject will be given a diary with questions about the preceding week. Reasons for non-adherence or no full adherence to the product will be reported, as well as adverse events, change in diet and co-medication, see Appendix 7 (Diary).

The diary will also include relevant telephone numbers, appointment details and other instructions and information.

8.3.3. FAQLQ Questionnaire

Food Allergy Quality of Life Questionnaire – Parent form 2-6 years will be used, see Appendic 3 (FAQLQ-PF). The FAQLQ is a disease specific instruments that evaluates the impact of food allergy on patients' health-related quality of life along 4 domains: allergen avoidance and dietary restrictions, emotional impact, risk of accidental exposure, and food allergy related health.

The FAQLQ is a 29-item questionnaire, self-administered and will be completed by the parent(s) during Visit 1 and when the subject has become tolerant to CM (i.e. after a negative DBPCFC) or during the final study visit in case of non-tolerance.

The total FAQLQ score is the mean score of all items with a range of 1 'no impairment' to 7 'maximal impairment'. Adding up the scores and dividing by the number of answered questions at each time point calculate the total FAQLQ score.

8.3.4. Skin prick tests

SPTs will be performed at visit 1, 2, 4 and 6, see also Appendix 8. In the SPT 1 negative control, 2 positive controls, CM and the GP product will be tested.

The SPTs will be performed by a trained allergy nurse according to the international standards. Fifteen minutes after the SPT, the contours of the reaction will be encircled with a fine-tip pen and transferred to a record sheet using transparent tape. Mean weal of the surface within the encircled area, the Histamine Equivalent Prick (HEP) index will be calculated. This will be done by dividing the area of the SPT of the allergen by the area of the mean of two histamines of the SPT using a scanning device (Hewlett Packard 2400c, Houston, TX, USA) and software that was earlier developed in the Erasmus MC hospital [www.PAAMOST.nl]. This method showed higher accuracy and reproducibility than the manually determined mean weal diameter (26).The cut-off value of 0.4 is used when interpreting the HEP-index. This corresponds to a weal size of 3 mm, the internationally accepted cut-off level for SPT for the mean weal diameter. This classification is a modification of the grading system described by Niemeijer et al. (27).

8.3.5. Blood samples

Blood samples will be drawn at visit 1 and at the end of the study or earlier when the child has become tolerant and intervention stops. Selected experiments in blood will be performed to study the mechanism of sensitization to casein and whey CM proteins using ISAC (Thermo Fischer B.V.). Specific samples will be stored for the measurement of genetic polymorphisms. Other assays will include immunoglobulin assays, and immune cell characterization assays. The allergens consist in two protein groups: Whey (20%, 5 g/L) and Casein (80%, 30 g/L). The following recombinant allergens are yet purified and characterized: Whey: Bos d 4: α-Lactalbumin, Bos d 5: β-Lactoglobulin, Bos d 6: Bovine serum albumin, Bos d 7: Immunoglobulins/ Lactoferrin. Whole casein: Bos d 9: αS1-casein, Bos d 10: αS2-casein, Bos d 11: β-Casein Bos d 12: κ-Casein. Hochwallner et al., 2014 (24).
Blood volume: <6 months of age 1 finger prick per occasion, ≥6 months of age 4 ml.

8.3.6. Double blind placebo controlled food challenge

The first DBPCFC is a standard diagnostic procedure and is not part of the study. However the data will be used for the study. This DBPCFC will be performed in accordance with PRACTALL guidelines (31). The medical staff should remain blinded until completion of the observation period of the second part of the challenge.

There are 3 sets of DBPCFC in this study, to be performed at visits 2 plus 3, 4 plus 5 and 6 plus 7.

Guidelines recommended for OFC for diagnosing Food Allergy (FA) will be followed. The DBPCFC is the diagnostic gold standard. (28). Because of the inherent risk of serious reactions, an OFC should be conducted at a medical facility with medical supervision and adequate emergency facilities and medication. The medical personnel should have experience in carrying out such challenges. Because of the risk of a severe reaction, intentional challenge should be avoided in patients who have recently experienced a life-threatening reaction to CM, particularly if it occurred more than once and if the patient is sensitised for CM.

The DBPCFC is carried out while the patient is on minimal or no symptomatic medication. Ideally the OFC starts with a low dose (lower than a dose that might induce a reaction) (29). While monitoring for any allergic symptoms, the dose is gradually increased, until a cumulative dose at least equivalent to a standard portion for age is consumed. The starting dose is typically in the range that produces an objective, but mild reaction for the most sensitive subjects. Studies also differ in challenge procedures, the form of the food used, the matrix in which the allergen was presented and the weight attributed to subjective and objective manifestations.

Efforts to standardize OFC began in 2004 with the position paper from the European Academy of Allergology and Clinical Immunology (30) and more recently the AAAAI and the EAACI published standardization of DBPCFC's with the PRACTALL guidelines (31).

In this study the Dutch guideline for CMA will be followed (18).The CM challenge is standardized with standard doses and a 30 minutes interval between each dose. The challenge material is dependent on the daily used eHF of the subject. In the Netherlands several different eHF's are used, from different industries, see Appendix 9. The various formulas have their own different challenge materials. For the study it is highly important to use the same challenge material per subject throughout the study. The eHF and the challenge material used in the initial diagnostic DBPCFC will be filed and added to the subject CRF and e-CRF.

Challenge material
Each child has its own food, that is continued during the entire study. The DBPCFCs will be performed with the same food. Challenge material will be added to the food. This test kit will be supplied ready for use by the manufacturer of that particular food. These are standard test kits that are in use in all relevant institutions in the Netherlands in standard dosages. For most formulas a specific test kit is available. Throughout the study the CM protein doses used for the challenges must be the same. However, the formula may change in order to match the formula that is used by the subject at that moment. Description of the different available kits (composition of the kit and the instructions for use) are available on the internet website of the manufacturer, see Appendix 4. A standardized protocol for CM challenges in either soy or rice milk is also added in Appendix 4.

Conduct of the DBPCFC
A regular breakfast before the DBPCFC is allowed. On the day of the DBPCFC the investigator completes the questionnaire on the medical feasibility / safety of the test, as presented in Appendix 17.

The personnel involved in the DBPCFC must be trained in the management of acute allergic reactions. The resuscitation equipment (including adrenaline for injection and oxygen) must be readily available. A crash cart must be available on site during all DBPCFCs and study product administrations.

Clinical symptoms and overall condition must be stable. Antihistamines must be discontinued at least 72 hours prior to the test.

Weight and height of the subject are measured and the exact doses of the drugs that might be necessary (rescue medication: adrenaline auto-injector (e.g. Epipen), adrenaline, clemastine, other antihistamines, xylometazoline and salbutamol) are computed and prepared:

Medication

Medication	Concentration	Dose	Administration
1. Adrenalin			
Adrenalin autoinjector	1 mg/ml	0.01 mg/kg = ml	i.m.
< 25 kg	0.15 mg/0.3 ml	0.15 mg	i.m.
> 25 kg)	0.30 mg/0.3 ml	0.30 mg	i.m.
2. Antihistamines			
Dimetindeen (Fenistil)	1 mg/ml	1 drop/kg = ml<br>max 20 drops	oral
Desloratadine (Aerius)	0.5 mg/ml	1.25 mg	oral
Clemastine (Tavegyl)	1mg/ml	0.025 mg/kg = ml<br>max 2 mg	i.m. / i.v.
3. Steroids			
Prednisolone	1 mg / ml	1 mg/kg = ml<br>(max 40 mg)	oral
4. Inhalation medication			

Salbutamol / Ipratropium bromide		< 4 yrs:<br>Salbutamol 2.5 mg / Ipratropium bromide 0.25 mg<br><br>> 4 yrs: Salbutamol 5.0 mg / Ipratropium bromide 2.5 mg	inhalation
Adrenaline	1 mg/ml	5 mg	inhalation

Randomization, blinding of the DBPCFC cow's milk
The placebo and active food challenges are performed on separate days with at least one-week interval in between. The sequence of the placebo and active test is randomized and blinded.

Assessment protocol for the outcome of the DBPCFC
Challenge sessions in which children consume less than 75% of the maximum challenge dose are considered invalid.

The challenge is discontinued when objective allergic symptoms occur, or subjective allergic symptoms occur twice on two successive administrations of the challenge material (29).

Objective signs and symptoms are: (angio)edema, urticaria, exacerbation of atopic eczema, vomiting, diarrhoea, lip or tongue swelling, rhino-conjunctivitis, stridor, coughing, wheezing, hoarseness, collapse, tachycardia and hypotension.

Subjective symptoms are defined as exacerbation of generalized itch (in case of atopic eczema), abdominal pain, nausea and/or cramp, oral allergy symptoms, itchy throat or sensation of throat swelling, difficulty in swallowing and other symptoms such as drowsiness and irritability. Immediate symptoms are defined as symptoms occurring during the challenge or within two hours after the last challenge dose.

A standardized scoring system will be used to score objective and subjective symptoms in the patients according to the PRACTALL guidelines. (31)

The subject may leave the hospital two hours after the last dose, when all symptoms (if any) have disappeared and the patient is in a stable condition.

Between 24 and 48 hours after the challenge a telephone call is made with the parent to enquire about late onset reactions. Late onset symptoms are defined as symptoms occurring between 2 and 48 hours after the last challenge dose (30). A standardized questionnaire is used to score late onset symptoms. The parents are advised to contact the hospital immediately in case symptoms at home occur.

Forty-eight hours after the second challenge session, the code is broken and the outcome of the DBPCFC is assessed as follows:

Active food challenge	Placebo	Assessment of DBPCFC
Positive (or clearly more positive/ more objective than placebo)	Negative	Positive
Positive	Positive	Negative
Questionable	Questionable	Negative
Negative	Negative	Negative
Negative (or positive, but clearly less positive than placebo)	Positive	Negative

During the 48 hours telephone call at 48 hours post challenge the parents are informed about the challenge result and the consequences.

Reschedule food challenge
In case the subject is ill, the DBPCFC should be postponed until the subject has recovered.

Follow-up protocol after a negative challenge at T= 2, 3 or 4
After a negative DBPCFC for CM the subject is assumed to be CM tolerant. In that case an introduction schedule with advice how to introduce the CM will be distributed. The parents of very young trial subjects will be advised to introduce CM in an increasing dose schedule added to the current formula. The parents of older subjects will be advised to introduce CM products to the daily diet of the child. After a symptom free introduction, the parents are advised to feed the subject 100 ml CM or another CM product e.g. yoghurt. After 2 months the research nurse will make a telephone call to check adherence to the schedule and to ask for symptoms during the introduction.

8.3.7. Microbiome measurements

Emerging evidence relates atopy and asthma to the composition and function of gut microbiota composition. Recent findings showed that food sensitization is associated with compositional changes in the gut microbiota. (32). There is an interaction with microbiota and food antigens, resulting in a sustained state of low-level physiological intestinal inflammation, protecting humans against severe infections but allows immunological tolerance to endogenous and foreign antigens. Our hypothesis is that alterations in human gut microbiota are associated with the development of food sensitization. Therefore, we are interested in possible changes in the gut microbiota of the children treated with the GP product in comparison with the placebo group.
At visit 1, 3, 5 and 7 the subject will be in the hospital for the full day. Samples of any stool will be collected in special containers (art. 727; DaklaPack Lelystad Europe BV) and will be stored at -80ºC as soon as possible. If no -80ºC freezer is available, storage at -20ºC for only a few weeks is acceptable. The samples will regularly be picked up in dry ice and transported to the Erasmus MC by an employer of the Erasmus MC. Sample's will be registered in Biobanking at the Erasmus MC.

8.3.8. Regular telephone calls throughout the study

All parents will be contacted by telephone monthly during the course of the study by the research or allergy nurse to assure that there are no safety or procedural issues. Parents will be able to ask questions and in case of problems an appointment with the investigator will be made.

8.4. Withdrawal of individual subjects

Parents can decide to leave the study at any time without any explanation. The investigator can decide to withdraw a subject from the study for medical reasons or in case of non-adherence to the study procedures.

A subject may be withdrawn from the study at any time.

Absolute withdrawal criteria are:

- Documented severe allergic reaction on test product.
- Study participation interferes with required medical treatment.
- Withdrawal of consent by both or one of the parents.
- Parent(s) of the subjects do not comply or are unable to comply with the study procedures.
- Geographic location that would limit compliance for scheduled visits.

8.5. Replacement of individual subjects after withdrawal

Withdrawn subjects will not be replaced.

8.6. Follow-up of subjects withdrawn from treatment

The CM tolerant children will enter a follow-up program consisting of regular telephone consults, using specific questionnaires on diet and allergic status.

Subjects withdrawn from treatment for other reasons (including adverse events and withdrawal of consent) will return to standard medical care.

8.7. Premature termination of the study

- The sponsor reserves the right to temporarily suspend or prematurely discontinue this study at any time for reasons of safety or ethical issues or severe non-compliance. For multicenter studies, this can occur at one or more or at all sites. The CCMO guideline on this subject will be respected in full.
- If the sponsor determines such action is needed, the sponsor will discuss the reasons for taking such action with the investigator or the head of the medical institution (where applicable).
- When feasible, the sponsor will provide advance notification to the investigator or the head of the medical institution, where applicable, of the impending action.
- If the study is suspended or prematurely discontinued for safety reasons, the sponsor will promptly inform all investigators, heads of the medical institutions (where applicable) and/or institution(s) conducting the study. The sponsor will also promptly inform the relevant regulatory authorities of the suspension or premature discontinuation of the study and the reason(s) for the action.

9. SAFETY REPORTING

9.1. Temporary halt for reasons of subject safety

In accordance to section 10, subsection 4, of the WMO, the sponsor will suspend the study if there is sufficient ground that continuation of the study will jeopardise subject health or safety. The sponsor will notify the accredited METC without undue delay of a temporary halt including the reason for such an action. The study will be suspended pending a further positive decision by the accredited METC. The investigator will take care that all subjects are kept informed.

9.2. AEs, SAEs and SUSARs

9.2.1. Adverse events (AEs)

Adverse events are defined as any undesirable experience occurring to a subject during the study, whether or not considered related to the experimental intervention. All adverse events reported spontaneously by the subject or observed by the investigator or his staff will be recorded.

9.2.2. Serious adverse events (SAEs)

A serious adverse event is any untoward medical occurrence or effect that

- results in death;
- is life threatening (at the time of the event);
- requires hospitalisation or prolongation of existing inpatients' hospitalisation;
- results in persistent or significant disability or incapacity;
- is a congenital anomaly or birth defect; or
- any other important medical event that did not result in any of the outcomes listed above due to medical or surgical intervention but could have been based upon appropriate judgement by the investigator.

An elective hospital admission will not be considered as a serious adverse event.

The investigator will report all SAEs to the sponsor without undue delay after obtaining knowledge of the events. .

The sponsor will report the SAEs through the web portal ToetsingOnline to the accredited METC that approved the protocol, within 7 days of first knowledge for SAEs that result in death or are life threatening followed by a period of maximum of 8 days to complete the initial preliminary report. All other SAEs will be reported within a period of maximum 15 days after the sponsor has first knowledge of the serious adverse events.

9.2.3. Suspected unexpected serious adverse reactions (SUSARs)

N.A.

9.3. Annual safety report

N.A.

9.4. Follow-up of adverse events

All AEs will be followed until they have abated, or until a stable situation has been reached. Depending on the event, follow up may require additional tests or medical procedures as indicated, and/or referral to the general physician or a medical specialist.
SAEs need to be reported till end of study within the Netherlands, as defined in the protocol.

9.5. Data Safety Monitoring Board (DSMB)

A DSMB will be utilized in this study to ensure external objective medical and/or statistical review of safety issues in order to protect the ethical and safety interests of subjects and to protect the scientific validity of the study. The DSMB is an independent committee. It is composed of a paediatrician-pulmonologist (chairman), 2 paediatricians (members) and a methodologist-statistician (member). These persons do not have any further involvement with the study.

The DSMB will oversee the safety aspects of the study as described in the DSMB charter.

The advice(s) of the DSMB will only be sent to the sponsor of the study. Should the sponsor decide not to fully implement the advice of the DSMB, the sponsor will send the advice to the reviewing METC, including a note to substantiate why (part of) the advice of the DSMB will not be followed.

The first assessment of safety will be performed after the 40th subject has been included or 4 months after inclusion of the 1st subject (whatever comes first). Thereafter assessments will be done every 4 months. However the DSMB may decide on an alternative frequency of meetings, based on the study information (e.g. safety data or enrolment results).

The DSMB will be provided guidelines on study halting criteria. While recommendations for stopping have been provided as a guide, the DSMB will base its recommendation to halt, or modify the study based on all available evidence. The guidance is not a substitute for the medical, scientific or statistical expertise of the DSMB body. The DSMB will receive all available safety data. The details of the schedule of planned interim analyses and the analysis plan for DSMB review is described in the charter which is available upon request.

10. DATA COLLECTION AND STATISTICAL ANALYSIS

10.1. Primary objective

Children who do not show clinical tolerance to CM with no more than mild symptoms will be considered treatment failures, as will subjects who fail to achieve and maintain the required daily dose of study product. Clinical tolerance to CM is defined as a negative DBPCFC with cow's milk, using the described assessment protocol in 6.7.4 for the outcome. An intent-to-treat analysis will be performed to test for a difference in tolerance rate. The analysis of the primary endpoint will involve the comparison of the proportion of children with CM tolerance at 24 months in the intervention and control groups. An univariable logistic regression model will be fitted with CM tolerance as outcome variable and treatment group as covariate. The odds ratio with 95% confidence interval will be used to assess the treatment effect.

The pace of the CMA resolution will be analysed with discrete time survival analysis. Time periods include 0 – 8 months; 9 – 16 months; 17 – 24 months. The data will be analysed with logistic regression in the person-period (long) format. The model includes time periods as categories, treatment group and the interaction between time periods and treatment group.

10.2. Secondary objectives

For all children, measurements of FAQLQ will be assessed at the beginning and end of the study (0, 8, 16 and 24 months). The treatment effect on the quality of life will be estimated with a linear regression model with quality of life at 24 months as outcome and treatment and quality of life at 0 months as covariates. The regression coefficient for treatment indicates the treatment effect. Confidence intervals will be assessed with bootstrapping, if the residuals of the regression model are not normally distributed.

Measurements of IgE to cow's milk will be assessed at the time points 0, 8, 16 and 24 months. This allows a comparison in change over time between the two treatment groups. We will analyze the difference with longitudinal multilevel mixed-effects regression models. The multilevel model will include random effects for the intercepts of the regression model and time coefficient of individual patients. The model will include fixed effects for treatment group and age. Focus will be on the interaction test between treatment group and time.

10.3 Other study parameters

N.A.

10.4 Interim analysis

A DSMB will review key safety data from the study. Enrollment in the study will continue during IDMC review. The IDMC will continue to review unblinded safety data as detailed in section 9.5 of this protocol about the DSMB.

No formal interim analyses of the safety data will be conducted, unless requested by the DSMB.

Detail of data to be reviewed and decision criteria will be included in the IDMC charter.

11. ETHICAL CONSIDERATIONS

11.1. Regulation statement

The study will be conducted in accordance with the principles of the current version of the Declaration of Helsinki, the guidelines for Good Clinical Practice and the Dutch Medical Research Involving Human Subjects Act (WMO).

11.2. Recruitment and consent

Patients will be recruited from the patient population of the participating hospital. When patients are eligible for the study (after a positive DBPCFC with cow milk), the treating physician will check the interest of parents and children for participation. Surrounding hospitals will be informed about the aim of the study and its inclusion criteria. Parents that are interested in the study can be referred to participating hospitals for more information and possible inclusion.

In case of potential interest the children and parents will be referred to the study staff for the formal informed consent procedure. In case the treating physician is also the investigator, another member of the study staff will handle the informed consent procedure. An independent physician will be available.

An informed consent form (ICF) will be provided to the parents. A consent form signed by both parents (or legal guardian) is mandatory for the participation of a child.

In case a potential study subject is presented without a documented positive DBPCFC (or a test performed more than 1 month ago), a prescreening phase may be introduced. This implies that only a DBPCFC is performed. If this test is positive, the subject may enter the screening phase of the iAGE study. A prescreening ICF is available.

Sufficient time will be allowed to consider participation, with a maximum of 2 weeks. This may be extended if deemed justified by the member of the study staff, who is in charge of the informed consent procedure.

11.3. Minors

All trial subjects will be minors up to 2 yaers of age. In case a minor does not cooperate or shows signs of resistance, the investigator should discontinue the study immediately. The guidelines of the Dutch Society for Pediatric Medicine (Nederlandse Vereniging voor Kindergeneeskunde (NVK)) for the protection of minor research subjects will be observed.

11.4. Benefits and risks assessment

11.4.1. Burden/risks:

- Total study duration: 2 years (or until development of tolerance);
- 7 visits (months 0, 4, 8, 12, 16, 20, 24);
- 6 phone calls to parents (between 24 and 48h after each challenge);
- 4 physical examinations (months 0, 8, 16, 24);
- 1 buccal swab (for genetic testing) from mother and child;
- 3-4 double blind placebo controlled food challenges (DBPCFC), each DBPCFC consists of 2 challenges (1 placebo, 1 verum, 1 week apart, randomized): during screening (in most clinics this challenge is part of standard care, in line with current guideline) and months 8, 16, 24 (in some clinics regular follow-up challenges are part of standard care as well, however for this study these 3 DBPCFC are considered study interventions);
- 4 skin prick tests (SPTs) (months 0, 8, 16, 24). Each SPT will consist of 5 pricks (CM, GP, 2 positive and 1 negative control);
- 4 blood samples (months 0, 8, 16 and 24 (or when tolerance has developed), blood volume: <6 months of age 1 finger prick per occasion, ≥6 months of age 4 ml;

- 4 stool samples (months 0,8, 16, 24), microbiome testing.
- Completion of questionnaires (months 0, 8, 16 and (FAQLQ only) 24), medical history child, parents, quality of life (FAQLQ);
- Interventional product: GP or placebo to be added to the regular daily nutrition of the subject starting at the first study day.
- The burden and risks will be minimal thanks to extensive experience with DBPCFCs. However, the outcome of the DBPCFC remains unpredictable and reactions may occur. Therefore, the DBPCFC will take place at the children's department, at the medium care unit, equipped with all necessary technical and medical emergency facilities.
- The study can only be performed in this group of children because high numbers of CMA are found in very young children.
- The DBPCFC is the only available diagnostic tool.
- Furthermore, this group is prone to develop tolerance and the window of tolerance induction is present in the first 2 years of the child.
- The above-described method is the golden standard for the diagnosis of CMA in children, according to the Dutch guideline.(18). For this study the DBPCFC is performed for an additional 3 times in order to measure any tolerance to CM during the intervention period.

11.4.2. Adverse Events

Children will be selected and included in the study after a positive DBPCFC for CM. This challenge is part of the food allergy protocol for the assessment of children with a suspicion of CMA. The study DBPCFCs will be performed in the clinic at the children's department. There is a validated protocol with controlled steps and all necessary rescue medication and medical staff in case of a reaction is at place. Treatment in case of an allergic reaction in according to the Dutch guidelines. Parents of subjects with an anaphylactic reaction during the DBPCFC or subjects with a moderate reaction to a low dose (step 1-4) in the DBPCFC will receive an adrenaline auto-injector. This auto-injector can be used at home in case of an anaphylactic reaction after accidental ingestion of CM. In this study a DBPCFC is performed 3 times (2 challenges per DBPCFC).
The first administration of the product (GP or placebo) will be done in the clinic. Up dosing of the product according to increasing weight of the child can be done at home.
SUSARs and SAEs will be reported to the ethics committee.

11.4.3. Additional Safety Monitoring and Risk Mitigation Details

Many safety measures have been already touched upon in the preceding section. Definitions of adverse events, ascertainment of relatedness and reporting requirements will not be described here in order to place emphasis within the space constraints on those details most directly impacting patient safety. With the report of moderate or severe symptoms occurring during home use of the GP product, the subject should be brought to the ER the day after the emergence of such symptoms for administration of the next dose of study product under medical supervision. If a dose administered at home is not have been tolerated, even on the basis of mild symptoms, the subject should also return to the Hospital for dosing under medical supervision. The recurrence of a mild symptom or symptoms over the course of several days of home use should suggest that the GP is not tolerated, even if each individual occurrence of symptoms could be assessed

Treatment of acute reactions should be with either an antihistamine and/or epinephrine, along with IV fluids, a beta-agonist (e.g., salbutamol, by inhaler or nebulizer), oxygen, and/or glucocorticosteroids, as indicated.

Mild acute allergic reactions can be transient and self-limiting, requiring no therapeutic intervention. Others, however, may require treatment. Generally, for mild symptoms requiring treatment, the subject should receive antihistamines.

Acute allergic reactions manifesting with moderate symptoms will generally require therapeutic

intervention, although some, even moderate, symptoms may on rare occasion be so transient that they do not require any specific treatment. Generally, for moderate symptoms requiring treatment, the subjects should receive antihistamines and/or epinephrine, as indicated. If there is uncertainty as to the severity of the reaction, administering epinephrine would be considered the most appropriate course of action.

11.4.4. Data and Safety Monitoring Plan.

Reactions, such as urticaria, flushing, oral pruritus, systemic pruritus, rhinitis, etc., will be monitored and recorded. The number of subjects requiring reduction or repeat administration of a dose due to mild symptoms will be examined.

Study treatment will be stopped if a subject has persistent clinical symptoms (gastro-intestinal (GI), respiratory or skin) at home after taking the daily dose of the study product. However, the subject remains in the study. The parent will be encouraged to attend all remaining study visits. The planned study procedures will be performed, with the exception of the DBPCFC and (subsequently) the 2 visits at each time point will be reduced to 1 visit.

If a subject has a severe systemic anaphylactic reaction defined as a multi-system reaction involving the skin, GI or respiratory tract and including hypotension or circulatory collapse, the study will be stopped and the protocol re-evaluated.

Safety of the subject will remain of primary importance. Any subject who develops severe systemic anaphylaxis, defined by any of the following: hypoxia (cyanosis or SpO2 <92% during reaction), documented hypotension (systolic blood pressure fall compared to baseline >30%), neurological compromise (confusion, unconsciousness), or incontinence after ingestion of the study product, significant hypotension during any stage of the protocol, and/or requires more than 2 injections of epinephrine during any administration of the GP product will be withdrawn from the study.

No interim analysis is planned.

All data collected in these studies will be retained in the research data files.

11.4.5. Potential Benefits

Potential benefits are:

3. Subjects may be desensitized to CM and have a reduced risk of anaphylaxis from an accidental ingestion of CM proteins.
4. Subjects may become tolerant to CM and do not have to avoid it.

However, subjects may not benefit.

The overall potential for benefit is favorable and risks are minimized as much as possible.

Potential benefits to society include a better understanding of food hypersensitivity reactions, better methods to treat food allergic individuals, and an improved understanding of food allergens involved in the human immune response.

11.4.6. Importance of the Knowledge to be gained.

Presently, the only effective treatment for CMA is avoidance. The primary goal of this study is to develop more effective CM formula for children with symptoms due to CM. This innovative application is designed to utilize emerging knowledge of the efficacy and safety of CM tolerance induction and to lower the risk of anaphylactic reactions and potentially cure the condition.

11.4.7. Risk assessment

1. Allergy prick skin testing: As this involves cutaneous exposure to an allergen extract, there is a potential risk of an allergic reaction, including itchy skin rash or hives, swelling, trouble

breathing, nausea, vomiting, and lower blood pressure. All of these are treated with topical steroids, with inhaled salbutamol, or, if necessary, with injections of epinephrine or other anti-allergic drugs. A trained member of the research team observes the subjects.

2. Taking blood: The risks of phlebotomy are minimal and include needle puncture, minor discomfort, bruising, or bleeding from the puncture site. Some subjects may also experience light-headedness or syncope, and these patients will be monitored.

Taking blood is generally more stressful for children. The option of the application of a crème with a local anesthetic ("Emla") exists.

3. Restricted Diet: The risk of nutritional deficiencies due to a restricted diet is minimized, by having a consultation with a registered dietitian. The dietician is also involved by the introduction of the CM after a negative CM challenge. Introduction schedules will be provided.

4. The challenges do carry a risk of an allergic reaction as they expose subjects to CM.

5. The use of the GP product: The risk of an allergic reaction during the use of the GP product is minimized by the very low dose of the product, which varies between 0,6 -1,5 gram/day dependent on the weight of the subject. At each time point the first up dosing intake takes place at the hospital.

11.4.8. Overall risk-benefit statement

The overall risk for the patient is minimal. The standard protocols for diagnostic procedures e.g. DBPCFC are followed, and will only be performed in presence of a physician, at the children's department of one of the participating hospitals. Standard usual care will continue. The GP product from Friesland Campina is judged by QA/QC to be compliant to the international guidelines and regulations for production and safety of IFT products. First dose will be given at the hospital in up dosing amounts, in presence of a physician.

11.5. Compensation

The sponsor/investigator has a liability insurance which is in accordance with article 7 of the WMO.

The sponsor (also) has an insurance which is in accordance with the legal requirements in the Netherlands (Article 7 WMO). This insurance provides cover for damage to research subjects through injury or death caused by the study.
The insurance applies to the damage that becomes apparent during the study or within 4 years after the end of the study.

Travel and parking expenses will be reimbursed in order to minimize expenses for the parents.

11.6. Incentives

There will be no incentives for participation. Travel and parking expenses will be reimbursed in order to minimize expenses for the parents. The subjects will be given a small present at the end of the study. This will not be mentioned in the information letter for the parent.

11.7. Storage of and access to study materials

All subjects who are eligible and for whom consent to participate has been obtained, will undergo allergen skin testing and a blood draw. Blood volume: <6 months of age 1 finger prick per occasion, ≥6 months of age 4 ml De-identified blood samples will be transported to the Wageningen University for further processing and storage. Plasma will be used for cow's milk specific immunoglobulin measurement (Phadia ImmunoCAP 100) and stored at -80°C for further proteomic analysis. Cells will be used for mechanistic studies, including the proposed work.

Clinical data will be maintained in the Erasmus MC database, which is accessible only to the

investigator and designated colleagues by individual passwords. All mechanistic studies using blood and plasma samples are de-identified, and the only individually identifiable information is stored in the ErasmusMC database. Information contained in the records may not be given to anyone unaffiliated with the study in a form that could identify them without the participants written consent, except as described in the consent form or as required by law. If the results of this study are published in a medical book or journal or used for teaching purposes, the names and other identifiers will not be used in any publication or teaching materials without the participant's specific written permission.

12. ADMINISTRATIVE ASPECTS AND PUBLICATION

12.1. Handling and storage of data and documents

The CRF will contain all the study related information of the patient including the completed questionnaires, results of SPT and ImmunoCAP and DBPCFC. The CRF will be stored at the site under the responsibility of the investigator. After the last visit the CRF will be sent to the coordinating investigator, and all data will be entered in the database. This database is kept under the responsibility of the coordinating investigator. A data manger will review the entries and will take care of back-up several times a day. Every subject is uniquely identified by the patient-ID, a combination of his/her centre number (see paragraph 4.2) and (sequential) patient number (001, 002 etc.). The local investigator keeps the code list.
The study documents and samples provided to the central study staff, central laboratories etc. will be coded with the patient-ID. Information revealing the patient's identity will not be given to study officials other than the local study team.
Study documents/data will be stored for 15 years after completion of the study. The blood samples will be kept until the study is concluded and will be destroyed thereafter.

12.2. Monitoring and Quality Assurance

A monitor from the Erasmus MC will visit each recruiting site at least once per 12 months. During a monitoring visit CRFs will be reviewed and source data will be compared to CRF data. All consent forms will be reviewed in full with regard to accuracy, completeness and adherence to applicable laws and guidelines.
Monitoring visits and all other relevant contacts with sites will be documented.
Appropriate QC tests will be performed on data entered in the database prior to data analysis.

12.3. Amendments

Amendments are changes made to the research after a favourable opinion by the accredited EC has been given. All substantial amendments will be notified to the EC that gave a favourable opinion.

Non-substantial amendments will not be notified to the accredited METC, but will be recorded and filed by the sponsor.

NB: The competent authority is in the Netherlands not involved in non-drug studies like these.

12.4. Annual progress report

The coordinating investigator will submit a summary of the progress of the trial to the accredited EC once a year. Information will be provided on the date of inclusion of the first subject, numbers of subjects included and numbers of subjects that have completed the trial, serious adverse events/ serious adverse reactions, other problems, and amendments.

12.5. Temporary halt and (prematurely) end of study report

The sponsor will notify the METC immediately of a temporary halt of the study, including the reason of such an action.

The investigator will notify the accredited EC of the end of the study within a period of 8 weeks. The end of the study is defined as the last patient's last visit. In case the study is ended prematurely, the investigator will notify the accredited EC within 15 days, including the reasons for the premature termination.

Within one year after the end of the study, the coordinating investigator will submit a final study report with the results of the study, including any publications/abstracts of the study, to the accredited EC.

12.6. Public disclosure and publication policy

An outline of the study protocol and a summary of the study results will be placed on the publically accessible website of the Dutch Trial Register (www.trialregister.nl).
The publication policy of the CCMO (March 2002, www.ccmo.nl) will be adhered to in full.

12.7. Existing infrastructure

The scientific challenges require a multidisciplinary approach and the input of two PhD students and one POSTDOC with different skills. Expertise is required on the following fields: in vitro IgE analysis, microbiome analysis, skin prick testing and DBPCFC, recognition and interpretation of clinical disease expression. This task is enormous and can be accomplished only by intensive collaboration between centres of excellence in this field. Ten clinics, experienced in the field of allergy in children, will carry out the clinical part of the study, whereas in vitro-analysis will be carried out in one central laboratory.

iAGE study team: Approx. 12 clinics will perform the clinical part of the study:

- Erasmus MC / Kinderhaven Rotterdam (centre 111)
- UMC Groningen (center 222)
- Noordwest Ziekenhuisgroep Alkmaar (center 555)
- Elkerliek Ziekenhuis Helmond (center 666)
- Canisius-Wilhelmina Ziekenhuis Nijmegen (center 777)
- Onze Lieve Vrouwe Gasthuis Amsterdam:(center 999)
- Zuyderland MC Heerlen (center 101)
- Catharina Ziekenhuis Eindhoven (center 102)
- Martini Ziekenhuis Groningen (center xxx)

NB: Changes in the list of participating centers (hospitals and/or principal investigators) will not result in a protocol amendment. Paragraph 4.2 of the protocol will be updated together with the next (non-) substantial protocol amendment.

Every hospital has a responsible paediatric allergist who coordinates the study and a research nurse who is in charge of the operational aspects of the study.

The iAGE study team has regular meetings, tentatively twice a year in March and November.

Each study team will be provided with questionnaires, challenge material, skin test material, and investigational product. An online randomisation protocol will be available for the clinics. As soon as a child meets the inclusion criteria, the team will enter the personal data in the randomisation program.

Responsibility for ImmunCAP measurements, e.g. recombinant allergens:
- Wageningen University.

Responsibility for the overall organization and database management:
- Erasmus MC Rotterdam, dept. Of Internal Medicine, section Allergology.

13. STRUCTURED RISK ANALYSIS

13.1. Potential issues of concern

13.1.1. Level of knowledge about mechanism of action

Well-known.

13.1.2. Previous exposure of human beings with the test product and/or products with a similar biological mechanism

Extensive exposure with test product in daily life and during clinical studies.

13.1.3. Can the primary or secondary mechanism be induced in animals and/or in ex-vivo human cell material

N.A.

13.1.4. Selectivity of the mechanism to target tissue in animals and/or human beings

N.A.

13.1.5. Analysis of potential effect

N.A.

13.1.6. Pharmacokinetic considerations

N.A.

13.1.7. Study population

Patients with cow's milk allergy. See also section 13.1.9.

13.1.8. Interaction with other products

Not expected.

13.1.9. Predictability of effect

In case of a severe reaction during the first DBPCFC, the risk of an allergic reaction during treatment is increased. This is the reason why the first administration of treatment is done in the clinic.

13.1.10. Can effects be managed?

Yes, with pharmaceuticals. See section 8.3.6.

13.2. Synthesis

Children will be selected and included in the study after a positive DBPCFC for CM. This challenge is part of the food allergy protocol for the assessment of children with a suspicion of CMA. The study DBPCFCs will be performed in the clinic at the children's department. There is a validated protocol with controlled steps and all necessary rescue medication and medical staff in case of a reaction is at place. Treatment in case of an allergic reaction in according to the Dutch guidelines. Parents of subjects with an anaphylactic reaction during the DBPCFC or subjects with a moderate reaction to a low dose (step 1-4) in the DBPCFC will receive an adrenaline auto-injector. This auto-injector can be used at home in case of an anaphylactic reaction after accidental ingestion of CM. In this study a DBPCFC is performed 3 times (2 challenges per DBPCFC).

The first administration of the product (GP or placebo) will be done in the clinic. Up dosing of the product according to increasing weight of the child can be done at home.
SUSARs and SAEs will be reported to the ethics committee.
Many safety measures have been already touched upon in the preceding section. Definitions of adverse events, ascertainment of relatedness and reporting requirements will not be described here in order to place emphasis within the space constraints on those details most directly impacting patient safety. With the report of moderate or severe symptoms occurring during home use of the GP product, the subject should be brought to the ER the day after the emergence of such symptoms for administration of the next dose of study product under medical supervision. If a dose administered at home is not have been tolerated, even on the basis of mild symptoms, the subject should also return to the Hospital for dosing under medical supervision. The recurrence of a mild symptom or symptoms over the course of several days of home use should suggest that the GP is not tolerated, even if each individual occurrence of symptoms could be assessed

Treatment of acute reactions should be with either an antihistamine and/or epinephrine, along with IV fluids, a beta-agonist (e.g., salbutamol, by inhaler or nebulizer), oxygen, and/or glucocorticosteroids, as indicated.

Mild acute allergic reactions can be transient and self-limiting, requiring no therapeutic intervention. Others, however, may require treatment. Generally, for mild symptoms requiring treatment, the subject should receive antihistamines.

Acute allergic reactions manifesting with moderate symptoms will generally require therapeutic intervention, although some, even moderate, symptoms may on rare occasion be so transient that they do not require any specific treatment. Generally, for moderate symptoms requiring treatment, the subjects should receive antihistamines and/or epinephrine, as indicated. If there is uncertainty as to the severity of the reaction, administering epinephrine would be considered the most appropriate course of action.

14. REFERENCES

1. Nwaru BI, Hickstein L, Panesar SS, Roberts G, Muraro A, Sheikh A, et al. Prevalence of common food allergies in Europe: a systematic review and meta-analysis. Allergy. 2014;69(8):992-1007.
2. Wood RA, Sicherer SH, Vickery BP, Jones SM, Liu AH, Fleischer DM, et al. The natural history of milk allergy in an observational cohort. J Allergy Clin Immunol. 2013;131(3):805-12.
3. Hilmenyuk T, Bellinghausen I, Heydenreich B, Ilchmann A, Toda M, Grabbe S, et al. Effects of glycation of the model food allergen ovalbumin on antigen uptake and presentation by human dendritic cells. Immunology. 2010;129(3):437-45. doi: 10.1111/j.365-2567.009.03199.x. Epub 2009 Nov 16.
4. Roth-Walter F, Berin MC, Arnaboldi P, Escalante CR, Dahan S, Rauch J, et al. Pasteurization of milk proteins promotes allergic sensitization by enhancing uptake through Peyer's patches. Allergy. 2008;63(7):882-90.
5. Stojadinovic M, Pieters R, Smit J, Velickovic TC. Cross-linking of beta-lactoglobulin enhances allergic sensitization through changes in cellular uptake and processing. Toxicol Sci. 2014;140(1):224-35.
6. Kim JS, Nowak-Wegrzyn A, Sicherer SH, Noone S, Moshier EL, Sampson HA. Dietary baked milk accelerates the resolution of cow's milk allergy in children. J Allergy Clin Immunol. 2011;128(1):125-31.e2. doi: 10.1016/j.jaci.2011.04.036. Epub May 23.
7. Ilchmann A, Burgdorf S, Scheurer S, Waibler Z, Nagai R, Wellner A, et al. Glycation of a food allergen by the Maillard reaction enhances its T-cell immunogenicity: role of macrophage scavenger receptor class A type I and II. J Allergy Clin Immunol. 2010;125(1):175-83 e1-11.
8. EFSA European Food Safety Authority PoDP, Nutrition and Allergies. Scientific Opinion on the essential composition of infant and follow-on formulae. EFSA Journal. 2014;12((7)):106 pp.
9. Nowak-Wegrzyn A, Bloom KA, Sicherer SH, Shreffler WG, Noone S, Wanich N, et al. Tolerance to extensively heated milk in children with cow's milk allergy. J Allergy Clin Immunol. 2008;122(2):342-7, 7 e1-2.
10. Feldman MF, Bird JA. Oral immunotherapy for food allergy, ready for prime time? Heated egg and milk. Curr Allergy Asthma Rep. 2014;14(5):436.
11. Wakayama J, Sugiyama S. Evaluation of temperature effect on the interaction between beta-lactoglobulin and anti-beta-lactoglobulin antibody by atomic force microscopy. Biochemistry. 2012;51(1):32-42.
12. Elofsson UM, Paulsson MA, Sellers P, Arnebrant T. Adsorption during Heat Treatment Related to the Thermal Unfolding/Aggregation of beta-Lactoglobulins A and B. J Colloid Interface Sci. 1996;183(2):408-15.
13. Shreffler WG, Wanich N, Moloney M, Nowak-Wegrzyn A, Sampson HA. Association of allergen-specific regulatory T cells with the onset of clinical tolerance to milk protein. J Allergy Clin Immunol. 2009;123(1):43-52 e7.
14. Ford LS, Bloom KA, Nowak-Wegrzyn AH, Shreffler WG, Masilamani M, Sampson HA. Basophil reactivity, wheal size, and immunoglobulin levels distinguish degrees of cow's milk tolerance. J Allergy Clin Immunol. 2013;131(1):180-6 e1-3.
15. Huang F, Nowak-Wegrzyn A. Extensively heated milk and egg as oral immunotherapy. Curr Opin Allergy Clin Immunol. 2012;12(3):283-92.
16. Petrus NC, Henneman P, Venema A, Mul A, van Sinderen F, Haagmans M, et al. Cow's milk allergy in Dutch children: an epigenetic pilot survey. Clinical and Translational Allergy. 2016;6(16).
17. Rachid R, Chatila TA. The role of the gut microbiota in food allergy. Current Opinion in Pediatrics. 2016;28(6):748-53.
18. Nederland' RwDvki. Richtlijn Diagnostiek van Koemelkallergie bij Kinderen in Nederland. 2012.

19. Greenhawt M, Fleischer D, Chan ES, Venter C, Stukus D, Gupta R, et al. LEAPing Through the Looking Glass: Secondary Analysis of the Effect of Skin Test Size and Age of Introduction on Peanut Tolerance after Early Peanut Introduction. Allergy. 2016.

20. DunnGalvin A, Cullinane C, Daly DA, Flokstra-de Blok BM, Dubois AE, Hourihane JO. Longitudinal validity and responsiveness of the Food Allergy Quality of Life Questionnaire - Parent Form in children 0-12 years following positive and negative food challenges. Clinical & Experimental Allergy. 2010;40(3):476-85.

21. Flokstra-de Blok BM, Dubois AE. Quality of life in food allergy: valid scales for children and adults. Current Opinion in Allergy and Clinical Immunology. 2009;9(3):214-21.

22. Sicherer SH, Sampson HA. Food allergy: Epidemiology, pathogenesis, diagnosis, and treatment. J Allergy Clin Immunol. 2014;133(2):291-307; quiz 8.

23. Sprikkelman AB, Heymans HS, Van Aalderen WM. Development of allergic disorders in children with cow's milk protein allergy or intolerance in infancy. Clin Exp Allergy. 2000;30(10):1358-63.

24. Hochwallner H, Schulmeister U, Swoboda I, Spitzauer S, Valentaa R. Cow’s milk allergy: From allergens to new forms of diagnosis, therapy and prevention. Methods. 2014;66(1):22-33.

25. Schoemaker AA, Sprikkelman AB, Grimshaw KE, Roberts G, Grabenhenrich L, Rosenfeld L, et al. Incidence and natural history of challenge-proven cow's milk allergy in European children--EuroPrevall birth cohort. Allergy. 2015;70(8):963-72.

26. van der Valk JP, Gerth van Wijk R, Hoorn E, Groenendijk L, Groenendijk IM, de Jong NW. Measurement and interpretation of skin prick test results. Clinical & Experimental Allergy. 2016;6(8).

27. Niemeijer NR, Fluks AF, de Monchy JG. Optimization of skin testing. II. Evaluation of concentration and cutoff values, as compared with RAST and clinical history, in a multicenter study. Allergy. 1993;48(7):498-503.

28. Boyce JA, Assa'ad A, Burks AW, Jones SM, Sampson HA, Wood RA, et al. Guidelines for the diagnosis and management of food allergy in the United States: summary of the NIAID-sponsored expert panel report. Nutrition Research. 2011;31(1):61-75.

29. Niggemann B, Beyer K. Diagnosis of food allergy in children: toward a standardization of food challenge. Journal of Pediatric Gastroenterology and Nutrition. 2007;45(4):399-404.

30. Bindslev-Jensen C, Ballmer-Weber BK, Bengtsson U, Blanco C, Ebner C, Hourihane J, et al. Standardization of food challenges in patients with immediate reactions to foods--position paper from the European Academy of Allergology and Clinical Immunology. Allergy. 2004;59(7):690-7.

31. Sampson HA, Gerth van Wijk R, Bindslev-Jensen C, Sicherer S, Teuber SS, Burks AW, et al. Standardizing double-blind, placebo-controlled oral food challenges: American Academy of Allergy, Asthma & Immunology-European Academy of Allergy and Clinical Immunology PRACTALL consensus report. Journal of Clinical Immunology. 2012;130(6):1260-74.

32. Chen CC, Chen KJ, Kong MS, Chang HJ et al. Alterations in the gut microbiotas of children with food sensitization in early life: Pediatr Allergy Immunol. 2016 May;27(3):254-62. doi: 10.1111/pai.12522. Epub 2016 Jan 21.

33. Leonard S et al. Baked Milk- and Egg-Containing Diet in the Management of Milk and Egg Allergy J Allergy Clin Immunol Pract 2015;3:13-23

34. Cherkaoui S. Powder milk: a user□friendly and safe product for heated□milk food challenge? Allergy Asthma Clin Immunol (2015) 11:39.

Appendix 01 – Flow chart

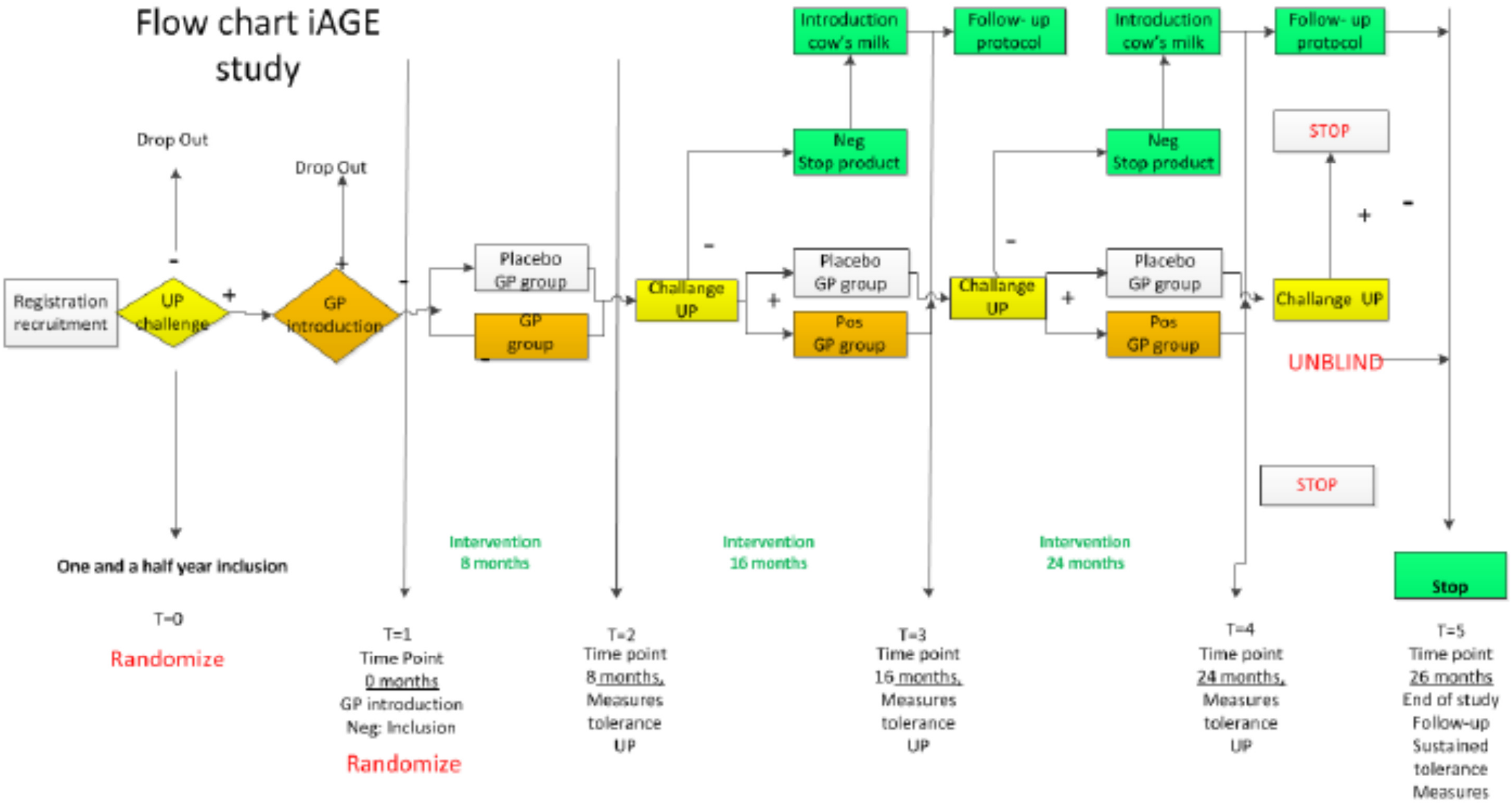

Appendix 02 – Study visits and assessments

Protocol scedule iAGE study	Standard DBPCFC CM				Visit 1			8 months visit					16 month visit					24 month visit			
Months 0,4,8,12,16,20,24 →					0 m		4m	8m		8m + 1w		12m	16m		16m + 1w		20m	24m		24m + 1 w	
challenges →	1db		2db					1db		2db			1db		2db			1db		2db	
					GP			UP		UP			UP		UP			UP		UP	
Time points →	T=0	48h		48h	T=1	48h		T=2	48h		48h		T=3	48h		48h		T=4	48h		48h
Visits→	V-2	☎	V-1	☎	V1	☎	V1a	V2	☎	V3	☎	V3a	V4	☎	V5	☎	V5a	V6	☎	V7	☎
Actions↓																					
Informed concent screening	x																				
Informed consent study					x																
Physical examination	x		x		x			x		x			x		x			x		x	
Case history mother, father, child					x																
Diary distribute (D) and/or collect ©*					D			CD					CD					C			
DBPCFC*	x		x					x		x			x		x			x		x	
48 hours telephone call*		x		x		x			x		x			x		x			x		x
SPT					x			x					x					x			
Blood sample (see 6.6 blood samples)					x			x					x					x			
FAQLQ-PF					x			x					x					x			
Result DBPCFC to parents*				x							x					x					x
Randomisation and establish dose product					x																
First dose test product (in hospital)					x																
Distribution test product*					x		x			x		x			x		x				
Microbioom feaces sample					x			x					x					x			
Genetics cheek swab chld and mother					x																
SIDE STUDY 1 (goat's milk, , see section protocol amendments)					x		x					x					x				
SIDE STUDY 2 (microbiome, see section protocol amendments)					x			x					x					x			

* NOT TO BE PERFORMED in case the child is tolerant

Appendix 03 – Questionnaire FAQLQ

FAQLQ-PF

De Voedselallergie
kwaliteit van leven vragenlijst
voor ouders van kinderen van 0-12 jaar

To cite the original Irish questionnaire:
DunnGalvin A, Flokstra-de Blok BMJ, Burks AW, Dubois AEJ, Hourihane JO. Food allergy QoL questionnaire for children aged 0-12 years: content, construct, and cross-cultural validity. Clin Exp Allergy 2008 Jun;38(6):977-986.

To cite the Dutch translated questionnaire:
van der Velde JL, Goossens NJ, DunnGalvin A, Hourihane JO, Duiverman EJ, Dubois AEJ, Flokstra-de Blok BMJ. Dutch parents report less impact of food allergy on health-related quality of life of their children (0-12 years) than Irish parents. Accepted by Clin Exp Allergy 2011.

Voedselallergie en kwaliteit van leven vragenlijst -

Versie voor ouders (FAQLQ-PF)

Kinderen van 0-12 jaar met een voedselallergie

Patient ID: ___ ____<br>(expl. 111 0001)

Initialen: ___ ___<br>(voorbeeld: NJ)

Geslacht□

Instructies voor ouders

- Hieronder volgen uitspraken van ouders over de invloed van voedselallergie op de kwaliteit van leven van hun kind.
- Geef per vraag aan hoeveel impact de uitspraak heeft op de **kwaliteit van leven van uw kind** door het juiste hokje aan te kruisen. De antwoorden zijn genummerd: 0-6.

Antwoordmogelijkheden

0 = Helemaal niet
1 = Bijna niet
2 = Enigszins
3 = Matig
4 = Nogal
5 = Erg
6 = Heel erg

- Als uw kind **0 tot 3 jaar** oud is, vul dan **Deel A, D** in.
- Als uw kind **4 tot 6 jaar** oud is, vul dan **Deel A , B, D** in.
- Als uw kind **7 jaar of ouder** is, vul dan **Deel A , B, C, D** in.

Deel A (0-12 jaar)

**Door de voedselallergie**	Helemaal niet **0**	**1**	**2**	**3**	**4**	**5**	Heel erg **6**
2 voelt mijn kind zich anders dan andere kinderen.	☐	☐	☐	☐	☐	☐	☐
3 baalt mijn kind van beperkingen in zijn/haar dieet.	☐	☐	☐	☐	☐	☐	☐
5 voelt mijn kind zich ongerust omdat ik bezorgd ben dat hij of zij een reactie op voeding zal krijgen.	☐	☐	☐	☐	☐	☐	☐

**Door de voedselallergie, …**	Helemaal niet **0**	**1**	**2**	**3**	**4**	**5**	Heel erg **6**
1 maakt mijn kind zich zorgen over voedsel	☐	☐	☐	☐	☐	☐	☐
4 is mijn kind bang om onbekende voedingsmiddelen te proberen.	☐	☐	☐	☐	☐	☐	☐
6 heeft mijn kind lichamelijke klachten.	☐	☐	☐	☐	☐	☐	☐
7 heeft mijn kind emotionele klachten.	☐	☐	☐	☐	☐	☐	☐
8 heeft mijn kind weinig variatie in zijn of haar dieet.	☐	☐	☐	☐	☐	☐	☐

**Voedselallergie heeft een negatieve invloed op mijn kind doordat…**	Helemaal niet **0**	**1**	**2**	**3**	**4**	**5**	Heel erg **6**
9 mijn kind meer aandacht krijgt dan andere kinderen van zijn of haar leeftijd.	☐	☐	☐	☐	☐	☐	☐
10 mijn kind sneller moest opgroeien dan andere kinderen van zijn of haar leeftijd.	☐	☐	☐	☐	☐	☐	☐
11 de omgeving van mijn kind beperkter is dan die van andere kinderen van zijn of haar leeftijd.	☐	☐	☐	☐	☐	☐	☐

**Door de voedselallergie is de sociale omgeving van mijn kind beperkt vanwege beperkingen in…**	Helemaal niet **0**	**1**	**2**	**3**	**4**	**5**	Heel erg **6**
12 restaurants waar we als gezin veilig naar toe kunnen gaan	☐	☐	☐	☐	☐	☐	☐
13 vakantiebestemmingen waar we als gezin veilig naartoe kunnen gaan.	☐	☐	☐	☐	☐	☐	☐

Helemaal niet → **Heel erg**

Door de voedselallergie zijn de mogelijkheden van mijn kind beperkt om deel te nemen aan…	0	1	2	3	4	5	6
14 sociale activiteiten in andermans huizen *(logeren, feestjes, spelen).*	☐	☐	☐	☐	☐	☐	☐

Deel B (4 tot 12 jaar)

Helemaal niet → Heel erg

Door voedselallergie zijn de mogelijkheden van mijn kind beperkt om deel te nemen aan…	0	1	2	3	4	5	6
15 activiteiten op de kleuterschool/school waarbij voedsel aanwezig is *(klassenfeest, traktaties, lunch).*	☐	☐	☐	☐	☐	☐	☐

Helemaal niet → Heel erg

Door voedselallergie	0	1	2	3	4	5	6
16 maakt mijn kind zich zorgen wanneer hij of zij naar nieuwe plaatsen gaat.	☐	☐	☐	☐	☐	☐	☐
17 baalt mijn kind omdat hij of zij altijd voorzichtig moet zijn met betrekking tot voedsel.	☐	☐	☐	☐	☐	☐	☐
18 voelt mijn kind zich buitengesloten bij activiteiten waar voedsel aanwezig is.	☐	☐	☐	☐	☐	☐	☐
19 vind mijn kind het vervelend dat bij familie uitjes *(uiteten, feestjes, dagje uit)* alles vooraf moet worden gepland.	☐	☐	☐	☐	☐	☐	☐
21 maakt mijn kind zich zorgen wanneer hij of zij met onbekende ouders/kinderen eet.	☐	☐	☐	☐	☐	☐	☐
22 baalt mijn kind van beperkingen op het sociale vlak.	☐	☐	☐	☐	☐	☐	☐

Helemaal niet → Heel erg

Door voedselallergie, …	0	1	2	3	4	5	6
20 is mijn kind bang om per ongeluk iets te eten waar hij of zij allergisch voor is.	☐	☐	☐	☐	☐	☐	☐
23 is mijn kind in het algemeen angstiger dan andere kinderen van zijn of haar leeftijd.	☐	☐	☐	☐	☐	☐	☐
24 is mijn kind in het algemeen bezorgder dan andere kinderen van zijn of haar leeftijd.	☐	☐	☐	☐	☐	☐	☐
25 is mijn kind niet zo zelfverzekerd in sociale situaties als andere kinderen van zijn of haar leeftijd.	☐	☐	☐	☐	☐	☐	☐
26 wenst mijn kind dat zijn of haar voedselallergie over zou gaan.	☐	☐	☐	☐	☐	☐	☐

- Als uw kind **0 tot 3 jaar** oud is, ga dan verder naar **Deel D**.
- Als uw kind **4 tot 6 jaar** oud is, ga dan verder naar **Deel D**.
- Als uw kind **7 jaar of ouder** is, vul dan **de hele vragenlijst in**.

Helemaal niet → **Heel erg**

Deel C (7-12 jaar)

	**Door voedselallergie**	0	1	2	3	4	5	6
27	voelt mijn kind zich bezorgd over zijn of haar toekomst (mogelijkheden, relaties).	☐	☐	☐	☐	☐	☐	☐
29	baalt mijn kind van slechte etikettering op verpakte voedselproducten.	☐	☐	☐	☐	☐	☐	☐

	**Beantwoord de volgende vragen**	0	1	2	3	4	5	6
28	Mijn kind heeft het gevoel dat veel mensen de ernst van voedselallergie niet begrijpen.	☐	☐	☐	☐	☐	☐	☐
30	Mijn kind heeft het gevoel dat zijn of haar leven wordt beperkt door voedselallergie in algemene zin.	☐	☐	☐	☐	☐	☐	☐

Deel D (0-12 jaar)

Beantwoord de volgende vragen alstublieft m.b.v. onderstaande antwoordmogelijkheden.

0	1	2	3	4	5	6
Nooit (0% kans)	Zeer kleine kans	Kleine kans	Redelijke kans	Grote kans	Zeer grote kans	Zeker (100 % kans)

	**Q1. Hoe groot is volgens u de kans dat…**	0	1	2	3	4	5	6
**1**	uw kind per ongeluk iets eet waar hij of zij allergisch voor is?							
**2**	uw kind een ernstige reactie krijgt wanneer hij of zij per ongeluk iets eet waar hij of zij allergisch voor is?							
**3**	uw kind overlijdt wanneer hij of zij per ongeluk iets eet waar hij of zij allergisch voor is?							
**4**	uw kind of mensen in de omgeving een allergische reactie **niet** goed kan/kunnen opvangen wanneer hij of zij per ongeluk iets eet waar hij of zij allergisch voor is?							

Onderstaande vragen lijken hetzelfde als bovenstaande vragen, maar het gaat er nu niet om dat u uw eigen mening weergeeft, maar dat u probeert om zonder overleg met uw kind de mening van uw kind weer te geven. We beseffen dat dit voor jonge kinderen moeilijk is, maar probeert u het alstublieft toch.

	**Q2. Hoe groot denkt u dat volgens uw kind de kans is dat..**	0	1	2	3	4	5	6
**1**	Hij/zij per ongeluk iets eet waar hij of zij allergisch voor is?							
**2**	Hij/zij een ernstige reactie krijgt wanneer hij of zij per ongeluk iets eet waar hij of zij allergisch voor is?							
**3**	Hij/zij overlijdt wanneer hij of zij per ongeluk iets eet waar hij of zij allergisch voor is?							
**4**	Hij/zij of mensen in de omgeving een allergische reactie **niet** goed kan/kunnen opvangen wanneer hij of zij per ongeluk iets eet waar hij of zij allergisch voor is?							

**Q4. Hoeveel voedingsproducten moet uw kind vanwege de voedselallergie vermijden?**	**Q5. Hoe groot is de invloed van de voedselallergie op het sociale leven van uw kind?**
☐ bijna geen	☐ verwaarloosbaar klein
☐ erg weinig	☐ zeer klein
☐ weinig	☐ klein
☐ sommige	☐ redelijk
☐ veel	☐ groot
☐ erg veel	☐ zeer groot
☐ bijna alle	☐ extreem groot

Appendix 04 – Recipe challenges CM various kits

1. Friso Allergy Care 1 provocatiekit (32)
(NB: Niet het doseerschema zoals vermeld op de provocatiekit gebruiken als dat afwijkt!)

Dosis 1: 0,05 ml testvoeding (Verum bevat ~1 mg Eiwit uit koemelk)
Tijdsinterval 20-30 minuten

Dosis 2: 0,2 ml testvoeding (Verum bevat ~3 mg Eiwit uit koemelk)
Tijdsinterval 20-30 minuten

Dosis 3: 0,7 ml testvoeding (Verum bevat ~10 mg Eiwit uit koemelk)
Tijdsinterval 20-30 minuten

Dosis 4: 2 ml testvoeding (Verum bevat ~30 mg Eiwit uit koemelk)
Tijdsinterval 30 minuten

Dosis 5: 7 ml testvoeding (Verum bevat ~100 mg Eiwit uit koemelk)
Tijdsinterval 30 minuten

Dosis 6: 21 ml testvoeding (Verum bevat ~300 mg Eiwit uit koemelk)
Tijdsinterval 30 minuten

Dosis 7: 71 ml testvoeding (Verum bevat ~1000 mg Eiwit uit koemelk)
Tijdsinterval 30 minuten

Dosis 8:
van 1-2 maanden: 69 ml totaal 171 ml (~2,4 g E) (restant 179 ml)
van 2-4 maanden: 90 ml totaal 192 ml (~2,7 g E) (restant 158 ml)
van 4-6 maanden: 112 ml totaal 214 ml (~3 g E) (restant 136 ml)
vanaf 6 maanden: 183 ml totaal: 285 ml (~4 g E) (restant 65 ml)

2. Nutramigen, Nutrilon Pepti en Neocate provocatiekit
(NB: Niet het doseerschema zoals vermeld op de provocatiekit gebruiken als dat afwijkt!)

Dosis 1: 0,1 ml testvoeding (Verum bevat ~1 mg Eiwit uit koemelk)
Tijdsinterval 20 minuten

Dosis 2: 0,2 ml testvoeding (Verum bevat ~3 mg Eiwit uit koemelk)
Tijdsinterval 20 minuten

Dosis 3: 0,75 ml testvoeding (Verum bevat ~10 mg Eiwit uit koemelk)
Tijdsinterval 20 minuten

Dosis 4: 2,25 ml testvoeding (Verum bevat ~30 mg Eiwit uit koemelk)
Tijdsinterval 30 minuten

Dosis 5: 7,75 ml testvoeding (Verum bevat ~100 mg Eiwit uit koemelk
Tijdsinterval 30 minuten

Dosis 6: 22,5 ml testvoeding (Verum bevat ~300 mg Eiwit uit koemelk)
Tijdsinterval 30 minuten

Dosis 7: 75 ml testvoeding (Verum bevat ~1000 mg Eiwit uit koemelk)
Tijdsinterval 30 minuten

Dosis 8:
van 1-2 maanden: 75 ml totaal 183 ml (2,4 g E)
van 2-4 maanden: 95 ml totaal 203 ml (2,7 g E)
van 4-6 maanden: 125 ml totaal 233 ml (3 g E)
vanaf 6 maanden: 192 ml Nutramigen (resterende totaal 300 ml met 4 g E voeding)
of 192 ml Nutrilon Pepti of Neocate (33 ml restant)

3. Recept provocaties koemelk in rijstemelk of sojamelk

Verumrecept
260 ml Rijstemelk (calcium verrijkt)
100 ml halfvolle gepasteuriseerde melk
40 ml limonadesiroop
12 g Bambix rijstebloem (blauw)

Rijstemelk en melk mengen, enigszins verwarmen en licht binden met Bambix.
Pas daarna de limonadesiroop toevoegen om schiften te voorkomen.

Placeborecept
260 ml Rijstemelk (calcium verrijkt)
6 g koemelkvrije margarine
40 ml limonadesiroop
12 g Bambix rijstebloem (blauw)

Rijstemelk enigszins verwarmen, margarine er door mengen en licht binden met Bambix.
Pas daarna de limonadesiroop toevoegen om schiften te voorkomen.

Doseerschema:
Dosis 1: 0,1 ml testvoeding (Verum bevat ~1 mg Eiwit uit koemelk)
Tijdsinterval 20-30 minuten

Dosis 2: 0,3 ml testvoeding (Verum bevat ~3 mg Eiwit uit koemelk)
Tijdsinterval 20-30minuten

Dosis 3: 1 ml testvoeding (Verum bevat ~10 mg Eiwit uit koemelk)
Tijdsinterval 20-30 minuten

Dosis 4: 3 ml testvoeding (Verum bevat ~30 mg Eiwit uit koemelk)
Tijdsinterval 30 minuten

Dosis 5: 10 ml testvoeding (Verum bevat ~100 mg Eiwit uit koemelk
Tijdsinterval 30 minuten

Dosis 6: 30 ml testvoeding (Verum bevat ~300 mg Eiwit uit koemelk)
Tijdsinterval 30 minuten

Dosis 7: 100 ml testvoeding (Verum bevat ~1000 mg Eiwit uit koemelk)
Tijdsinterval 30 minuten

Dosis 8: 259,6 ml voeding (Verum bevat ~2556 mg Eiwit uit koemelk)
2 uur observatie tijd

Totaal: 404 ml voeding met ~4 g Eiwit uit koemelk

Appendix 05 – Dosing of product

Het GP en placebo product

Het product wordt geleverd in blik. De ouders krijgen afhankelijk van het gewicht van het kind een maatschepje, waarmee 1 maal per dag een afgestreken schepje van het product aan de voeding van het kind wordt toegevoegd.
Elke 3 maanden zou het kind 1 kilo groeien, dus een hogere hoeveelheid moeten toedienen. De kinderen krijgen voor 4 maanden materiaal mee. Na deze 4 maanden bezoekt de ouder het ziekenhuis kort voor het wegen van het kind en het ophalen van een tweede blik met nieuw schepje afhankelijk van het gewicht van het kind, voor de volgende 4 maanden. Hierbij wordt het volgende schema gebruikt:

Child		Proteïne intake	Total proteïne intake	5% GP or placebo treatment	
Age (months)	Weight (kg)	Grams/kg body weight	Grams/day	Grams/day	4 diff amounts
3	6	2 gr/kg	12	0.6	0.8
6	7	2 gr/kg	14	0.7	0.8
9	8	2 gr/kg	16	0.8	0.8
12	9	2 gr/kg	18	0.9	1.1
15	10	2 gr/kg	20	1	1.1
18	11	2 gr/kg	22	1.1	1.1
21	12	2 gr/kg	24	1.2	1.4
24	13	2 gr/kg	26	1.3	1.4
30	14	2 gr/kg	28	1.4	1.4
36	15	2 gr/kg	30	1.5	1,7
48	17	2gr/kg	34	1,7	1,7

Bij visit 1 (na randomisatie) wordt het studie materiaal wat klaarstaat voor de ouders om mee te nemen, in oplopende dosering toegediend, om evt. reacties te kunnen monitoren. De nurse weegt het af in de juiste hoeveelheid. De nurse legt ook hoe het schepje gebruikt dient te worden.

	Doses mg						Cum
GP product (mg)	10	30	100	300	1000	2000	3440
ml eHF	0,6	1,7	5,8	17,4	58,1	116,3	200

Appendix 06 – Questions bi-monthly telephone call

Vragen 2 maandelijkse bellijst, iAge studie

1. Drinkt uw kind over het algemeen de flessen met het studieproduct leeg?

○ Ja ○ Nee

2. Hoe vaak per week drinkt uw kind gemiddeld de flessen niet leeg?

○ Nooit ○ 1x/week ○ 1-3x/week ○ >3x/week

3. Indien uw kind de flessen niet leeg drinkt wat is dan de reden?

○ Wil niet verder drinken ○ Spuugt het uit

4. Zijn er reacties geweest op het studie middel?

○ Ja ○ Nee

5. Zo ja welke reactie?

○ Uitslag van de huid/toename eczeem

○ Galbulten

○ Zwelling van de lippen en/of ogen

○ Gastrointestinale klachten met buikpijn en/of diarree

○ Kortademigheid

○ Wegraking

6. Vergeet u het studieproduct weleens te geven?

○ Nee, nooit ○ 1x/week ○ 1-3x/week ○ >3x/week

7. Is er een allergische reactie geweest op een ander middel?

○ Ja ○ Nee

8. Zo ja welke reactie?

○ Uitslag van de huid/toename eczeem

○ Galbulten

○ Zwelling van de lippen en/of ogen

○ Gastrointestinale klachten met buikpijn en/of diarree

○ Kortademigheid

○ Wegraking

9. Welk product?

○ Kippenei ○ Pinda ○ Tarwe ○ Noten ○ Vis ○ Schaaldieren ○ Anders, nl

Appendix 07 – Diary for parents

iAGE week dagboek

DATUM : ………../………../……………

(dit is altijd een datum op zondag, u vult de afgelopen week in)
In dit dagboek vermeldt u gegevens over de afgelopen week. Een week duurt van maandag tot en met zondag.
In de eerste tabel wordt gevraagd of uw kind de fles met het product helemaal heeft leeggedronken. Als dit het geval is kunt U een kruisje zetten in deze kolom. Indien uw kind de fles niet helemaal of helemaal niet heeft leeggedronken kunt u een kruisje zetten in de 2e kolom. In de 3e kolom vragen we U op de hoeveelheid te noteren die hij/zij heeft laten staan. In de 4e kolom vragen we u op te schrijven waarom de fles niet volledig is leeggedronken.
Soort voeding: ………………………………………….. Aantal voedingen per dag: ……….. x Hoeveelheid voeding per keer: ……….……… ml

Dag van de week	Fles met product is helemaal leeggedronken	Fles met product is niet helemaal opgedronken	Noteer hoeveelheid die hij/zij heeft laten staan (ml)	Wat is de reden waardoor de fles niet helemaal leeggedronken is?
Maandag				
Dinsdag				
Woensdag				
Donderdag				
Vrijdag				
Zaterdag				
Zondag				

De volgende tabel gaat over eventuele klachten die uw kind de afgelopen week heeft gehad. Graag vermelden waar uw kind last van had.

Dag van de week	Klachten (huid, maag/darmen, luchtwegen, keel/neus/oor, urinewegen, koorts etc…)	Andere klachten zoals bijvoorbeeld veel huilen, slecht slapen, onrustig zijn, overstrekken
Maandag		
Dinsdag		
Woensdag		
Donderdag		
Vrijdag		
Zaterdag		
Zondag		

De laatste tabel gaat over het medicijngebruik van uw kind. Wilt u de medicijnen die het kind heeft gebruikt invullen. Mocht het medicijn en de dosis en de reden elke dag hetzelfde zijn, dan mag u dat bij de maandag invullen en bij de andere dagen een aanhalingsteken gebruiken.

Dag van de week	Naam medicijn(en)	Dosis van medicijn: hoe vaak per dag en hoeveelheid per keer vermelden (bv 1 x per dag 5 ml)
Maandag		
Dinsdag		
Woensdag		
Donderdag		
Vrijdag		
Zaterdag		
Zondag		

Dank u wel voor het invullen. Indien u nog aanvullende opmerkingen heeft mag u die op de achterkant van het formulier schrijven.
ACHTERZIJDE
Bijzonderheden:

Produkten gegeten die (mogelijk) koemelk bevatten (zie voedingslijst achterin dit dagboek):
Wanneer (noteer dag en tijdstip):
Wat gegeten (bv koekjes van Verkade):
Hoeveel gegeten (bv 1/4^e koekje):
Reactie: ja/nee
Zo ja beschrijf aard en tijdstip van reactie:

Vragenlijst maken voor dietiste / doktersassistente / student / / PhD student / kinderarts: elke 2 mnd vragen of voeding lukt (compliance), reacties?, koemelkprodukten
Dietiste: lijst maken met produkten waar koemelk in zit
Lijst moet bij het dagboek zitten

Appendix 08 – Skin prick test form

SPT formulier

Patient ID: ___ ____
(expl. 111 0001)

Initialen: ___ ___
(voorbeeld: NJ)

Geslacht

Positive controle	GP product		
Positive Controle			
Negative controle			
Koemelk			

Appendix 09 – Types of formulas in NL

Soort voeding	Naam voeding	Fabrikant
Intensief gehydrolyseerd obv wei-eiwit	Nutrilon Pepti<br>Friso Pep	Nutricia<br>CampinaFriesland
Intensief gehydrolyseerd obv caseine-eiwit	Nutramigen LGG<br>Friso Allergy Care	Mead Johnsson<br>CampinaFriesland
Intensief gehydrolyseerd obv vrije AZ	NeoCate<br>Nutramigen PURAMINO	Nutricia<br>Mead Johnsson
Kunstvoeding obv soja-eiwit	Nutrilon Soya	Nutricia
Overig	Rijstemelk	

Neocate LCP (< 1 jr)
Neocate Spoon (> 6 mnd)
Neocate Junior (> 1 jr)
Neocate Active en Neocate Advance (> 1 jaar; gaat uit de handel, hiervoor Neocate Junior in de plaats)
Nutrilon Pepti 1 (< 6 mnd); 41 % lactose
Nutrilon Pepti 2 (> 6 mnd); 36 % lactose
Nutrilon Pepti jr (oudere kinderen)

Nutramigen LGG 1 (< 6 mnd); lactosevrij
Nutramigen LGG 2 (> 6 mnd); lactosevrij
Nutramigen PURAMINO

Friso Allergy Care 1 (< 24 mnd); lactosevrij
FrisoPep 1 (< 6 mnd); bevat lactose
FrisoPep 2 (6 – 12 mnd); bevat lactose

Protocol amendment 01 – side study goats milk sensitization in cow's milk allergic infants

PROTOCOL AMENDMENT 01	
GOATS MILK SENSITIZATION IN COW'S MILK ALLERGIC INFANTS	
ADDENDUM TO THE PROTOCOL	
**Date**:	29-January 2018
**Applicable to site(s)**:	All
**Type of amendment**:	Substantial amendment<br>Side study<br>Goat milk added to all 4 skin pick tests

RATIONALE

Nowadays, goat's milk (GM) consumption has been re-evaluated for its potential benefits to human health. Furthermore, goat's milk in virtue of its higher content in short chain, medium chain, mono and polyunsaturated fatty acids, is more digestible than cow's milk (CM).(1) It contains the highest amount of oligosaccharides among domestic animals, while GM oligosaccharides show significant similarities to human milk oligosaccharides from a structural point of view. (2)

As goat's or sheep's milk (GSM) proteins are highly homologous to CM proteins, clinical cross-reactivity is expected and IgE sensitization to GSM proteins has been found to be as high as 92 to 98% in children with IgE-mediated CM allergy (CMA) (3). The high cross-reactivity between milk from goats, sheep, and cows described in literature means that goat's and sheep's milk is prohibited in CMA patients. Conversely, CM tolerant individuals should also tolerate GSM. However, several cases of GSM allergy without CMA have been regularly reported.(4, 5)

Allergenicity of infant formulas based on GM was studied in 26 Italian infants and children (aged 5 months to 7 years) who were allergic to CM proteins. All subjects showed positive skin test responses to both CM and GM; in a double-blind, placebo-controlled food challenge, 26 of 26 children reacted to CM and 24 of 26 reacted to GM. (3)

Little is known about tolerance to GSM in patients receiving CM oral immune therapy (CMOIT); however, there are very few published case reports of allergy to GSM in CM tolerant and also in patients after OIT with CM. (6) Del Rio et al. found a high prevalence (26%) of allergy to GSM in a population of CMA children treated with oral immunotherapy. Therefore, tolerance to GSM should be assessed in order to provide accurate nutritional advice and minimize life-threatening accidental intake. Specific IgE and Skin Prick test (SPT) to goat's and sheep's whole milk is a good marker of this allergy.(6)

The information provided above has resulted in the addition of a sub study to the iAGE study with the following objectives:

PRIMARY OBJECTIVE

- What is the effect of tolerance induction with CM on the sensitization rate in SPT to GM?

SECONDARY OBJECTIVES

- What is the percentage of cross sensitization in SPT to GM in Dutch children with CM allergy.

- Is there a relation between the time to reach tolerance to CM and the sensitization rate (in SPT) to GM in CM allergic children.

ADDITIONAL STUDY TESTS FOR PARTICIPANTS TO THR SIDE STUDY
In the main study 4 SPTs have been scheduled: visits 1, 2, 4 and 6. A GM allergen will be added to the existing 5 “pricks” at all 4 visits.
The procedure for the SPTs as described in section 8.3.4 of the study protocol will be adhered to.

CONDUCT OF THIS SIDE STUDY
This side study will be conducted in all study sites. The parents of all potential trial subjects will be asked for their informed consent for this side study as well.
This side study was originally published as a separate document for the time being. It was included in the full protocol at the time of the protocol update 3.0 dated 23-07-2018.
The written information for the parents about this side study was included in the ICF.

REFERENCES
1. Jirillo F, Magrone T. Anti-inflammatory and anti-allergic properties of donkey's and goat's milk. Endocr Metab Immune Disord Drug Targets. 2014;14(1):27-37.
2. Kiskini A, Difilippo E. Oligosaccharides in goat milk: structure, health effects and isolation. Cell Mol Biol (Noisy-le-grand). 2013;59(1):25-30.
3. Restani P. Goat milk allerginicity. J Pediatr Gastroenterol Nutr. 2004;39(4):323-4.
4. Hazebrouck S, Ah-Leung S, Bidat E, Paty E, Drumare MF, Tilleul S, et al. Goat's milk allergy without cow's milk allergy: suppression of non-cross-reactive epitopes on caprine beta-casein. Clin Exp Allergy. 2014;44(4):602-10.
5. Blanc F, Bernard H, Alessandri S, Bublin M, Paty E, Leung SA, et al. Update on optimized purification and characterization of natural milk allergens. Mol Nutr Food Res. 2008;52 Suppl 2:S166-75.
6. Rodriguez del Rio P, Sanchez-Garcia S, Escudero C, Pastor-Vargas C, Sanchez Hernandez JJ, Perez-Rangel I, et al. Allergy to goat's and sheep's milk in a population of cow's milk-allergic children treated with oral immunotherapy. Pediatr Allergy Immunol. 2012;23(2):128-32.

Protocol amendment 02 – side study microbiome in cow's milk allergic infants

PROTOCOL AMENDMENT 02

MICROBIOME SIDE STUDY IN COW'S MILK ALLERGIC INFANTS

ADDENDUM TO THE PROTOCOL

**Date**:	29-January 2018 (updated 23-July-2018)
**Applicable to site(s)**:	All
**Type of amendment**:	Substantial amendment<br>Side study<br>Microbiome and food allergy

RATIONALE

In recent findings food allergy appear to be associated with alterations in the gut microbiota or dysbiosis early in life, that may be predictive of disease persistence versus tolerance acquisition and contribute to the manifestation of atopy and allergy in children (1). Gut microbiota is found to play a critical role in the regulation of the early immune system, which can influence allergy development (2). The infant immune system is not fully developed and the gut microbiota may provide the first signal for maturation of a balanced postnatal innate and adaptive immune system and T helper (Th)1/Th2 cell response.
The link between gut microbiota and food allergy is extensively studied, but not yet fully clarified. The natural history between different food allergies is distinct, therefore specific microbiota may be associated with each food allergen. Ling et al. showed a correlation between specific faecal microbial species in the infant and IgE-mediated food allergy (3). A recent multi-center longitudinal study of 226 children with cow's milk allergy examined the relationship between the gut microbiome and allergy resolutions and found that Firmicutes, including Clostridia, were enriched in the early infant gut microbiome of patients whose milk allergy resolved by age 8 years (4).
Furthermore, cow milk allergy is a multifactorial disease, triggered by both genetic and environmental factors. Our understanding of allergic diseases has been advanced by data generated through genome-wide association studies (GWAS) (https://www.genome.gov/gwastudies/). Most GWAS are performed in asthma and peanut allergy (5, 6). Marenholtz et al. reported a GWAS on OFC-proven food allergy which stratified the results for the three most common food allergens and no validated loci for CMA is found (7). However, if a genetic susceptible locus is found, the biologic relevance of this variant is sometimes not clear. Within the Dutch EuroPrevall birth cohort study, Petrus et al. aimed to detect epigenetic associations specific within CMA (8). They found methylation differences in regions of genes involved in immunological pathways and associated with other allergies. However, the statistical power is limited (n=20 CMA). Integrating genotype data with transcriptional profiles can prioritize causal variation, as genetic variants associated with RNA transcript changes in disease tissue, are more likely to be relevant than those that do not affect transcript levels. Therefore, in this study with 200 cow's milk allergic infants, saliva and blood samples will be collected and stored for further (epi)genetic analysis.
It has been shown that part of the microbiome is heritable, implicating that host genetics are an important factor in determining gut microbiome composition (9). Twin studies indicated that the microbiome can be an important mediator between host genetics and phenotype. Different heritable bacteria were identified (10). However, the effect of host genetics on the gut microbiome is likely masked by environmental and exogenous factors. Therefore, large sample sizes are necessary and the analysis of the gut microbiome in relation to the host genetics remains challenging (11).

Furthermore, besides the gut, other sites of the human body are also colonized with site specific microbiota (12). The skin microbiome has been studied in relation to atopic dermatitis (AD). AD is associated with food allergy and the estimated prevalence of AD in infants of age < 1 year with cow's milk allergy is 40% (13). AD is a chronic, inflammatory skin disease with symptoms including erythema, scaling and intense itching, which cause significant impairment in quality of life (14). Previous work has demonstrated that S. aureus colonisation is adversely correlated with disease severity of AD patients, 70% of lesional and 39% of nonlesional skin sites are colonized by S. aureus. AD patients more often showed an IgE antibody response directed against S. aureus superantigens compared to healthy controls (15). Furthermore, S. aureus not only colonizes the skin and mucosae, the most frequent carriage site is the anterior nares of the nose (31). Studies have also indicated that the nasal colonization is associated with AD prevalence and severity in infants (32). Furthermore, S. aureus can cause IgE mediated sensitisation and directly induce degranulation of mast cells through δ-toxins (16,17). Other changes in the skin microbiome are also identified, with low bacterial diversity at lesional sites and decrease of specific species during flares (18,19).

Environmental factors are known to manipulate microbiome composition. Some of these factors include duration of gestation, time of weaning, feeding after delivery, mode of delivery, early-life antibiotic use and infant hospitalization (20). Data of these factors in our study population, will be collected through questionnaires and taken into account in the analysis. By integrating data on multiple scales including genetics, microbial and environmental entities, we tend to develop a systems biologic approach which is a necessary step in our development of a more complete understanding of CMA and allergic diseases.

Of these environmental factors, feeding plays a major role in the development of the infant gut microbiome. Breast milk is composed of different nutrients and bioactive factors, that support the development and maturation of the infant gut and the innate and adaptive immune system (21). In a recently published 12-month longitudinal study of 107 healthy mother-infant pairs, breastfed infants received 27.7% of their gut bacteria from breast milk during the first month of life (22). Bacterial diversity and composition changes were associated with the proportion of daily breast milk intake in a dose-dependent manner, even after introduction of solid foods. Oligosaccharides in breast milk contain growth factors that are beneficial for bacteria such as Bifidobacterium (23). Formula-fed infants were more often colonized with Escherichia coli, Clostridium difficile, Bacteroides and lactobacilli (24). Recent evidence indicated that mixed feeding (human milk and formula) which is most often seen in common practice, resulted in gut microbiota that is similar to exclusively formula-fed infants (25).

Furthermore, direct relations between breast milk components and epigenetic changes are found (26). The effects of breast milk on DNA methylation, could be mediated through the gut microbiome. Breast milk also contains microRNAs (miRNAs), involved in gene expression regulation at the post-transcriptional level. Within the EuroPrevall birth cohort study, hypomethylation of the EIF4E2 region was identified in CMA infants (8). EIF4 protein family plays a key role in the mTORC1 signaling pathway and Melnik et al. reported the importance of breastmilk in this pathway and in early development (27). The finding of hypomethylation of EIF4E2 and dysregulation of the mTORC1 pathway in CMA infants, might be the result of absence of maternal miRNAs. To conclude, different factors highlight the value of investigating the role of breast milk in relation to the gut microbiome in CMA infants.

Thus, early infancy could be the key window of opportunity for microbial effects on early immune system development and cow's milk allergy development. Increased gut microbial richness at age 3 months is associated with decreased food sensitization by age 1 year (24). Furthermore, Bunyavanich et al. found that gut microbiome composition at age 3 to 6 months, but not 7 to 12 or 13 to 16 months, is associated with CMA resolution (8). These findings support the notion that microbial effects on early immune system development play a role in food allergy and in CMA development in specific. Therefore, in able to move to a systems biology approach of CMA and study the early immune system, we aim to investigate the gut microbiome in infants with proven CMA, while taken into account the human genome and environmental factors of influence.

The information provided above has resulted in the addition of a sub study to the iAGE study. We aim to better understand the nature and resolution of CMA, by integrating knowledge of the microbiome into a systems biology context of the study subjects and environment. We collect and analyze the stool samples in infants age 3 months to 2 years, at 4 different time-points within the study period of 2 years This will allow us to move beyond associations between the microbiota and allergy outcomes. This approach will contribute to and will build on precision medicine for allergy care.

PRIMARY OBJECTIVES
- Identify characteristics of the gut microbiome composition in CM allergic infants.
- Investigate additional role of the gut microbiome in CMA tolerance induction in infants.

SECONDARY OBJECTIVES
- Identify breast milk microbial composition and consequent colonization of gut microbiota in CM allergic infants.
- Study genetic markers associated with CMA.
- Investigate the role of the gut microbiome in the development of atopic disorders in CM allergic infants.
- Additional information in stratification of patients with atopic dermatitis and cow's milk allergy through analysis of S. aureus colonisation via culture and alterations in the microbiome.

ADDITIONAL STUDY TESTS FOR PARTICIPANTS TO THR SIDE STUDY
Stool: At visit 1, 3, 5 and 7 the subject will be in the hospital for the full day. Samples of any stool will be collected in special containers and will be stored at - 80ºC as soon as possible.
NB: If no - 80ºC freezer is available, storage at - 20ºC for only a few weeks is acceptable.

Skin: 2 swabs for culture of S. aureus and 2 swabs for microbiome analysis will be taken of the skin, one area with eczema and one area without. The swab will be rubbed over the skin site for 30 seconds, two swabs for each area of at least 4 cm2.
The swab samples for culture will be sent to the department of Microbiology and a semi quantitative culture technique for identification of S. aureus will be performed. The swab samples for microbiome analysis will be stored at -80ºC as soon as possible. If no -80ºC freezer is available, storage at -20ºC for only a few weeks is acceptable. The samples will regularly be picked up in dry ice and transported to the Erasmus MC by an employer of the Erasmus MC. Samples will be registered in Biobanking at the Erasmus MC. The samples will be analyzed and the relative abundance of all present species in de samples will be characterized using 16S rRNA sequencing.

Nasal: 2 swabs for culture of S, aureus will be taken from the anterior nares of the nose at visits 1, 3, 5 and 7. The swab samples for culture will be sent to the Dept. of Microbiology and a semi-quantitative culture technique for identification of S. aureus will be applied.

Human milk: Detailed information on breastfeeding will be obtained from parental questionnaires during the first visit. Breast milk is collected using manual pumping into 20ml non-sterile bottles. These bottles will be stored at -20ºC directly. We estimated that within the study population, 25% of the mothers will be breastfeeding during 2 different time points (n=100).

Saliva: Oragene [DNA Genotek OG-250] is a non-invasive sample collection method that yields large amounts of high quality DNA from a 2-mL saliva sample that is stable for several years at room temperature. DNA from both infant and mother will be collected (n=400) and purified and processed on the Illumina® BeadChip 610Quad assay. Instructions stated on the Oragene DNA collection kit are followed.

SAMPLE SIZE

The primary objective of the iAGE study focuses at the effect of the GP treatment on CM tolerance induction at 8, 16 and 24 months. Based on a previous study, it is expected that 33% of children in the control group will be CM tolerant after 24 months and in the intervention group 56% (33) Using an alpha of 0.05 (2-sided) and 90% power, 93 children per group will be required. The aim is to recruit at least 200 (100 per study arm) participating children (age 3 months – 2 years.) with a proven CMA, (positive double-blind placebo-controlled food challenge [DBPCFC] to CM) (34). In this study this number should be sufficient to measure tolerance in 8, 16 or 24 months. Stool is collected on the time points of T=1, 2, 3, and 4. Therefore 800 samples will be available for performing analysis. We estimate that 25% of the mothers will be breastfeeding (non-exclusively), during two different time point of the study. This equals to 100 samples of breast milk.

SAMPLE ANALYSIS AND STATISTICAL ANALYSIS

Bacterial DNA from the stool and breast milk samples will be isolated. The V4 region of the 16S rRNA gene is amplified with barcoded primers and 16S rRNA sequencing is performed on the Illumina MiSeq platform using 2 x 250 bp paired-end read. We will perform sequence data analysis of the microbiome using Quantitative Insights into Microbial Ecology (QIIME) 1.8.0, an open-source bioinformatics pipeline. Operational taxonomic units (OTUs) will be constructed using a more than 97% similarity threshold. Alfa and beta diversity are measured. Principal coordinate analysis (PCoA) will be used to visualize clustering patterns between samples based on beta diversity distances.

Human DNA is isolated from saliva samples. DNA methylation profiles will be obtained using… array of Illumina. Raw data is processed and analyzed with statistical analysis scripted in ''R''. The presence of technical and/or biological bath effects is estimated and evaluated by principal component analysis (PCA). QQ-plots will be generated. Statistical analysis on individual probe differentially methylated positions (DMPs) and on specific annotated differentially methylated regions (DMR) within a gene will be performed.

CONDUCT OF THIS SIDE STUDY

This side study will be conducted in all study sites. The parents of all potential trial subjects will be asked for their informed consent for this side study as well.

This side study was originally published as a separate document for the time being. It was included in the full protocol at the time of the protocol update 3.0 dated 23-07-2018.

The written information for the parents about this side study was included in the ICF.

REFERENCES

1. Rachid R, Chatila TA. The role of the gut microbiota in food allergy. Current Opinion in Pediatrics. 2016;28(6):748-53.
2. Gensollen, Thomas et al. Correlation between early-life regulation of the immune system by microbiota and allergy development. Journal of Allergy and Clinical Immunology , Volume 139 , Issue 4 , 1084 – 1091.
3. Ling Z, Li Z, Liu X, Cheng Y, Luo Y, Tong X, Yuan L, Wang Y, Sun J, Li L, Xiang C. Altered fecal microbiota composition associated with food allergy in infants. Appl Environ Microbiol. 2014 Apr;80(8):2546-54. doi: 10.1128/AEM.00003-14. Epub 2014 Feb 14.
4. Bunyavanich S, Shen N, Grishin A, Wood R, Burks W, Dawson P, Jones SM, Leung DYM, Sampson H, Sicherer S, Clemente JC. Early-life gut microbiome composition and milk allergy resolution. J Allergy Clin Immunol. 2016 Oct;138(4):1122-1130. doi: 10.1016/j.jaci.2016.03.041. Epub 2016 May 10.
5. Torgerson DG, Capurso D, Mathias RA, Graves PE, Hernandez RD, Beaty TH, et al. Resequencing candidate genes implicates rare variants in asthma susceptibility. Am J Hum Genet 2012;90:273-81
6. Brown, S. J. et al. Loss-of-function variants in the filaggrin gene are a significant risk factor for peanut allergy. J. Allergy Clin. Immunol. 127, 661–667 (2011).
7. Marenholz I et al., Genome-wide association study identifies the SERPINB gene cluster as a susceptibility locus for food allergy. Nat Commun. 2017 Oct 20;8(1):1056. doi: 10.1038/s41467-017-01220-0.

8. Nicole C. M. Petrus, Peter Henneman, Andrea Venema , Adri Mul, Femke van Sinderen, Martin Haagmans, Olaf Mook, Raoul C. Hennekam, Aline B. Sprikkelman and Marcel Mannens. Cow's milk allergy in Dutch children: an epigenetic pilot survey.
9. Turnbaugh, P.J. et al. (2009) A core gut microbiome in obese and lean twins. Nature 457, 480–484.
10. Goodrich, J.K. et al. (2016) Genetic determinants of the gut microbiome in UK twins. Cell Host Microbe 19, 731–743.
11. Host Genetics and Gut Microbiome: Challenges and Perspectives, Alexander Kurilshikov et al, Volume 38, Issue 9, September 2017, Pages 633-647.
12. The Human Microbiome Project Consortium*; Structure, function and diversity of the healthy human microbiome; Nature 486, 207-214 (14 June 2012) doi: 10.1038/nature11234.
13. Novembre E, Vierucci A; Milk allergy/intolerance and atopic dermatitis in infancy and childhood; Allergy 2001;56 Suppl 67:105-8.
14. Weidinger S, Novak N; Atopic dermatitis; The Lancet Volume 387, Issue 10023, 12–18 March 2016, Pages 1109-1122.
15. de Wit J, Totté JEE, van Buchem FJM, Pasmans SGMA; The prevalence of antibody responses against Staphylococcus aureus antigens in patients with atopic dermatitis: a systematic review and meta-analysis; Br J Dermatol. 2017 Dec 16. doi: 10.1111/bjd.16251.
16. Sonesson A, Bartosik J, Christiansen J, et al. Sensitization to skin-associated microorganisms in adult patients with atopic dermatitis is of importance for disease severity. *Acta Derm Venereol;* 2013; 93: 340–45.
17. Nakamura Y, Oscherwitz J, Cease KB, et al. *Staphylococcus* δ-toxin induces allergic skin disease by activating mast cells. *Nature* 2013; 503: 397–401.
18. Dybboe R, Bandier J, Skov L, Engstrand L, Johansen JD. The Role of the Skin Microbiome in Atopic Dermatitis: A Systematic Review. Br J Dermatol. 2017.
19. Kong HH, Oh J, Deming C, Conlan S, Grice EA, Beatson MA, et al. Temporal shifts in the skin microbiome associated with disease flares and treatment in children with atopic dermatitis. Genome Res. 2012;22(5):850-9.
20. Aymé Spor, Omry Koren, Ruth Ley Unravelling the effects of the environment and host genotype on the gut microbiome. *Nature Reviews Microbiology* 9, 279–290 (2011) doi:10.1038/nrmicro2540.
21. O'Sullivan A, Farver M, Smilowitz JT. The Influence of Early Infant-Feeding Practices on the Intestinal Microbiome and Body Composition in Infants. Nutrition and Metabolic Insights. 2015;8(Suppl 1):1-9. doi:10.4137/NMI.S29530.
22. Association Between Breast Milk Bacterial Communities and Establishment and Development of the Infant Gut Microbiome, Pannarajai et al. *JAMA Pediatr.* 2017;171(7):647-654. doi:10.1001/jamapediatrics.2017.0378.
23. Goldsmith, F., O'Sullivan, A., Smilowitz, J.T. et al. J Mammary Gland Biol Neoplasia (2015) 20: 149. https://doi.org/10.1007/s10911-015-9335-2.
24. Bäckhed F, Roswall J, Peng Y, Feng Q, Jia H, Kovatcheva-Datchary P, Li Y, Xia Y, Xie H, Zhong H, Khan MT, Zhang J, Li J, Xiao L, Al-Aama J, Zhang D, Lee YS, Kotowska D, Colding C, Tremaroli V, Yin Y, Bergman S, Xu X, Madsen L, Kristiansen K, Dahlgren J, Wang J. Dynamics and Stabilization of the Human Gut Microbiome during the First Year of Life. Cell Host Microbe. 2015 Jun 10;17(6):852. doi: 10.1016/j.chom.2015.05.012. Epub 2015 Jun 10.
25. Juliette C. Madan, MD, MS ;Anne G. Hoen, PhD ;Sara N. Lundgren, BA; Association of Cesarean Delivery and Formula Supplementation With the Intestinal Microbiome of 6-Week-Old Infants *JAMA Pediatr* .219-212:(3)170;2016 ،doi:10.1001/jamapediatrics.2015.3732.
26. Verduci E et al. Epigenetic effects of human breast milk. Nutrients. 2014 Apr 24;6(4):1711-24. doi: 10.3390/nu6041711.
27. Bodo C. Melnik, Excessive Leucine-mTORC1-Signalling of Cow Milk-Based Infant Formula: The Missing Link to Understand Early Childhood Obesity, J Obes. 2012; 2012: 197653.
28. Azad MB[1], Konya T, Guttman DS, Field CJ, Sears MR, HayGlass KT, Mandhane PJ, Turvey SE, Subbarao P, Becker AB, Scott JA, Kozyrskyj AL; CHILD Study Investigators. Infant gut microbiota and food sensitization: associations in the first year of life. Clin Exp Allergy. 2015 Mar;45(3):632-43. doi: 10.1111/cea.12487.
29. Kim JS, Nowak-Wegrzyn A, Sicherer SH, Noone S, Moshier EL, Sampson HA. Dietary baked milk accelerates the resolution of cow's milk allergy in children. J Allergy Clin Immunol. 2011;128(1):125-31.e2. doi: 10.1016/j.jaci.2011.04.036. Epub May 23.
30. Sicherer SH, Sampson HA. Food allergy: Epidemiology, pathogenesis, diagnosis, and treatment. J Allergy Clin Immunol. 2014;133(2):291-307; quiz 8.
31. Williams REO. Healthy carriage of Staphylococcus aureus: its prevalence and importance. Bacteriol Rev 1963: 27: 56-71.

32. Lebon A. Labout JAM, Verburgh HA, et al. Role of Staphylococcus aureus Nasal Colonization in Atopic Dermatitis in Infants The Generation R Study. Arch Pediatr Adolesc Med. 2009; 163(8):745-749. Doi:10.1001/archpediatrics.2009.11
